# Validity of Inertial Measurement Units to Measure Lower-Limb Kinematics and Pelvic Orientation at Submaximal and Maximal Effort Running Speeds

**DOI:** 10.3390/s23239599

**Published:** 2023-12-04

**Authors:** Yi-Chung Lin, Kara Price, Declan S. Carmichael, Nirav Maniar, Jack T. Hickey, Ryan G. Timmins, Bryan C. Heiderscheit, Silvia S. Blemker, David A. Opar

**Affiliations:** 1 School of Behavioural and Health Sciences, Australian Catholic University, Fitzroy, VIC 3065, Australia; kara.price@acu.edu.au (K.P.); declan.carmichael@acu.edu.au (D.S.C.); nirav.maniar@acu.edu.au (N.M.); jack.a.hickey@mu.ie (J.T.H.); ryan.timmins@acu.edu.au (R.G.T.); david.opar@acu.edu.au (D.A.O.); 2 Sports Performance, Recovery, Injury and New Technologies (SPRINT) Research Centre, Australian Catholic University, Fitzroy, VIC 3065, Australia; 3 Department of Sport Science and Nutrition, Maynooth University, W23 F2H6 Co. Kildare, Ireland; 4 Department of Orthopedics and Rehabilitation, University of Wisconsin-Madison, Madison, WI 53705, USA; heiderscheit@ortho.wisc.edu; 5 Department of Biomedical Engineering, University of Virginia, Charlottesville, VA 22908, USA; ssblemker@virginia.edu; 6 Springbok Analytics, Charlottesville, VA 22902, USA

**Keywords:** gait analysis, IMU, inertial sensors, optical motion capture, running mechanics, root mean square error, Bland–Altman analysis, statistical parametric mapping, biomechanical model

## Abstract

Inertial measurement units (IMUs) have been validated for measuring sagittal plane lower-limb kinematics during moderate-speed running, but their accuracy at maximal speeds remains less understood. This study aimed to assess IMU measurement accuracy during high-speed running and maximal effort sprinting on a curved non-motorized treadmill using discrete (Bland–Altman analysis) and continuous (root mean square error [RMSE], normalised RMSE, Pearson correlation, and statistical parametric mapping analysis [SPM]) metrics. The hip, knee, and ankle flexions and the pelvic orientation (tilt, obliquity, and rotation) were captured concurrently from both IMU and optical motion capture systems, as 20 participants ran steadily at 70%, 80%, 90%, and 100% of their maximal effort sprinting speed (5.36 ± 0.55, 6.02 ± 0.60, 6.66 ± 0.71, and 7.09 ± 0.73 m/s, respectively). Bland–Altman analysis indicated a systematic bias within ±1° for the peak pelvic tilt, rotation, and lower-limb kinematics and −3.3° to −4.1° for the pelvic obliquity. The SPM analysis demonstrated a good agreement in the hip and knee flexion angles for most phases of the stride cycle, albeit with significant differences noted around the ipsilateral toe-off. The RMSE ranged from 4.3° (pelvic obliquity at 70% speed) to 7.8° (hip flexion at 100% speed). Correlation coefficients ranged from 0.44 (pelvic tilt at 90%) to 0.99 (hip and knee flexions at all speeds). Running speed minimally but significantly affected the RMSE for the hip and ankle flexions. The present IMU system is effective for measuring lower-limb kinematics during sprinting, but the pelvic orientation estimation was less accurate.

## 1. Introduction

The ability to run at the maximal or near-maximal speed is a critical requirement for many sporting activities and an important assessment following injury rehabilitation [1]. Optical motion capture (OMC) is the most common technique in the literature for studying running mechanics. An OMC system uses multiple high-speed cameras and reflective or optoelectronic markers placed on anatomical landmarks to accurately measure joint angles and segmental motions during running [2,3]. While OMC has been used extensively for more than two decades [4], its practical utility is limited because of the requirement of a complex infrastructure of cameras, markers, and controlled laboratory environments.

In contrast to OMC, inertial measurement unit (IMU) systems offer great versatility and have emerged as a prominent tool for collecting kinematics in various activities, without being restricted to the laboratory environment [5,6]. An IMU typically consists of accelerometers and gyroscopes that measure linear acceleration and angular rate of the attached object, respectively. Some IMUs also have a magnetometer to enhance the orientation estimation by detecting the magnetic north direction. The popularity of IMU systems has led to a growing number of studies assessing their validity and reliability in analysing walking and running kinematics [6,7]. Researchers have validated IMUs against OMC for estimating hip, knee, and ankle joint angles in the sagittal plane during moderate-speed running (up to 5 m/s) with a root mean square error (RMSE) of less than 10° [6]. Studies have also shown the validity of IMU systems compared with force plate-based running velocity [8] and OMC-based trunk kinematics [9] during sprinting. Nevertheless, there is still a lack of detailed knowledge regarding the validity of IMUs in assessing lower-limb kinematics during maximal and near-maximal speeds.

Researchers have employed various statistical measures to assess the accuracy of discrete and continuous variables computed by IMU systems during running [7,10]. A Bland–Altman analysis was commonly used to evaluate the agreement between IMU measurements and reference systems (e.g., OMC) by quantifying differences in discrete parameters (such as peak joint angles) [11,12]. To investigate differences in joint kinematics throughout the entire stride cycle, researchers typically calculate the RMSE and Pearson correlation coefficient (*r*). Recently, a statistical parametric mapping (SPM) analysis has been used to identify specific regions in the gait cycle where IMU-based measurements differ significantly from those obtained from the reference system [13,14,15]. An SPM allows for a comparison of the entire time-varying, one-dimensional biomechanical data (e.g., kinematic trajectories) both in magnitude and shape. To the best of our knowledge, no study has employed an SPM analysis to identify regions within the time-continuous curves, where significant differences in measurements occur between IMU and OMC systems during maximal speed running.

The primary objective of the present study was to conduct a detailed evaluation of the accuracy of IMU measurements compared to OMC-based measurements during maximal and near-maximal running. Our specific focus was on determining the IMU system’s ability to provide accurate lower-limb joint angles in the sagittal plane and pelvic orientation (pelvic tilt, obliquity, and rotation) at running speeds ranging from 70 to 100% of the maximal running speed. Considering that previous research reported an RMSE of IMU-based sagittal plane joint angles below 10° during moderate-speed running [6], we hypothesized that the RMSE between the OMC and IMU systems would also remain below 10° during high-speed running. Additionally, due to the reported speed dependency of IMU-based thigh kinematics during sprinting [16], we hypothesized that the accuracy of IMU-based measurements would also be running speed dependent.

## 2. Materials and Methods

### 2.1. Participants

Twenty adults participated in this study (14 males, 6 females; age: 26.8 ± 5.5 years; height: 174.5 ± 8.7 cm; mass: 74.3 ± 11.2 kg; BMI: 24.1 ± 2.8 kg/m^2^). Each participant was recreationally active and free from any musculoskeletal injuries that could have a negative impact on their sprinting performance. The sample size employed in our study aligns with that reported in several review papers on IMU validation [6,17,18]. Each participant provided informed written consent after ethics approval was granted by the Human Research Ethics Committee, Australian Catholic University (2021-194H).

### 2.2. Data Collection

Running data were collected at the Biomechanics Laboratory on ACU’s Melbourne campus. All experiments were carried out by the same two trained operators to minimize inter-operator variation. Prior to the actual data collection, each participant underwent a familiarisation session to ensure their comfort and familiarity with running at four specific speeds (70%, 80%, 90%, and 100% of their maximal speed) on a curved non-motorized treadmill (Curve 3, Woodway USA, Inc., Pewaukee, WI, USA). The Pacer Performance software version 2 (Pacer Performance system, Innervations., Perth, Australia) was used to collect and display real-time running speed to help participants maintain the target running pace accordingly for submaximal trials.

Data collection was conducted on the same non-motorized treadmill one week after the familiarisation session. Prior to collecting the required data, a warm-up procedure, including walking, jogging, and running at 80% of the perceived maximal effort, was employed on the treadmill for each participant. The data acquisition began with participants standing stationary for 10 s to gather anthropometric measurements for developing participant-specific musculoskeletal models. Subsequently, participants were asked to run at their perceived maximal speed (100%), followed by three submaximal speeds of 70%, 80%, and 90% of the maximal speed achieved during the 100% trial, in incremental order. The real-time running speed measured by the Pacer Performance software was used as a visual aid to ensure each participant’s running speed was as close as possible to the target speed. Each participant performed two trials at each running speed, and each trial started with a synchronisation task by making a hard stomp with their right foot on the treadmill. A well-defined peak knee flexion angle created during the stomp was used for time synchronisation in post-processing. Participants were required to complete a minimum combined total of 10 consecutive stride cycles (comprising five right-limb and five left-limb stride cycles) while maintaining their target speeds for each trial. To reduce fatigue, each participant was provided with a minimum of two minutes of rest between trials.

**Figure 1:**
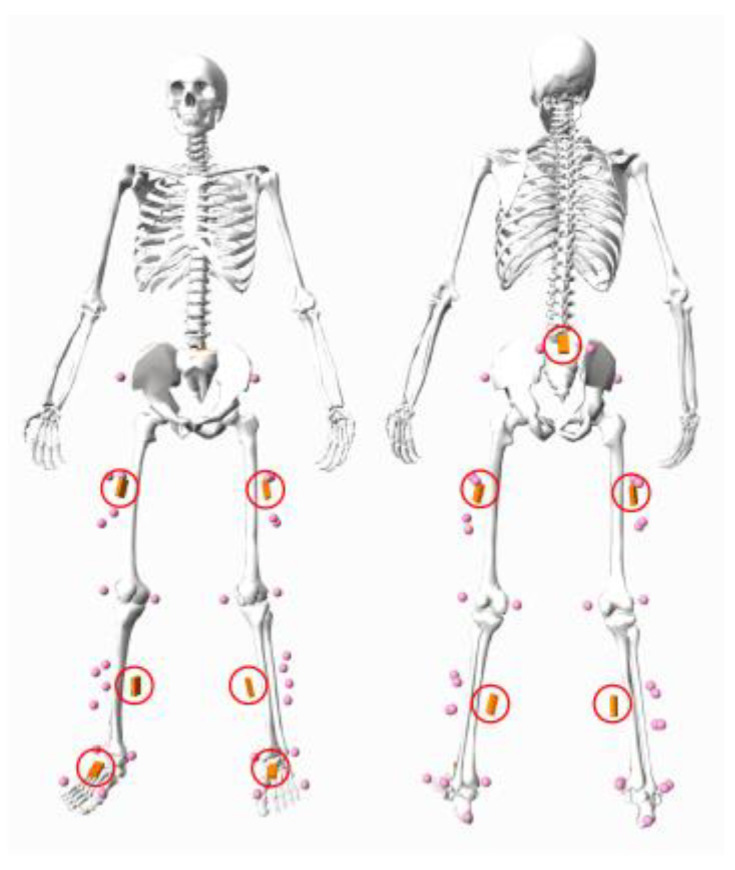
A biomechanical model in standing pose showing the experimental marker set (pink spheres) and IMU positions (circled in red).

Joint motion data were collected simultaneously using a 10-camera OMC system (Vicon, Oxford Metrics, Oxford, UK) and a seven-IMU motion capture system (Xsens MVN Awinda system, Movella, Enschede, The Netherlands). Thirty retroreflective markers (14 mm in diameter) were mounted on the pelvis and both lower limbs according to our previous work [19,20,21,22] with trajectories captured by the OMC system at 200 Hz (pink spheres in Figure 1). One IMU was placed over each participant’s sacrum, and three IMUs were placed on the lateral aspect of mid-thigh, medial aspect of mid-shank, and dorsal aspect of mid-foot, respectively, for each leg (red circles in Figure 1). Each IMU was affixed using Velcro straps (Xsens MVN Awinda system, Movella, Enschede, The Netherlands) and equipped with an accelerometer, gyroscope, and magnetometer to measure linear acceleration, angular acceleration, and magnetic heading, respectively, in a local three axe sensor frame [23]. The IMU system recorded data at a 100 Hz sampling rate.

Both the OMC and IMU systems underwent a standardised calibration process prior to data collection. For the OMC system, we used the Active Wand (Vicon, Oxford Metrics, Oxford, UK) to calibrate the cameras and set up the reference coordinate system. In the case of the IMU system, a recommended N-pose calibration was used to calibrate the IMU sensors for each participant [23].

### 2.3. Biomechanical Modelling

Participant-specific biomechanical models were created by scaling a generic model available in OpenSim (version 3.3) [24]. The pelvis of this model was connected to the ground via a six degrees of freedom (DOF) joint (three translations and three rotations). Each leg was represented by five segments, which were actuated by a 3-DOF ball-and-socket hip joint, a 1-DOF hinge knee joint, a 1-DOF hinge ankle joint, a 1-DOF hinge subtalar joint, and a 1-DOF hinge metatarsal joint. Both the subtalar and metatarsal were locked in the present study. The anthropometric measurements collected from each participant’s static standing trial were used to scale the generic model using OpenSim’s scale tool. Specifically, the tool was used to determine segment-dependent scale factors by comparing marker distances measured on the segment during the static standing trial to the corresponding distances between virtual markers on the generic model. These scaling factors were subsequently applied to adjust segment lengths and segment inertial properties.

### 2.4. OMC-Based Joint Angles

The inverse kinematic algorithm available in OpenSim was used to compute OMC-based joint angles for all running trials. During each instant of the stride cycle, the angles of the joints were calculated by minimizing the sum of the squared differences between the positions of virtual markers on the participant-specific model and experimental marker trajectories [25].

### 2.5. IMU-Based Joint Angles

IMU-based joint angles were obtained directly from the proprietary manufacturer software Xsens MVN Analyze 2022.0 (Movella, Enschede, The Netherlands). A two-step process implemented in MVN Analyze was used to convert the three-sensor modulus (accelerometer, gyroscope, magnetometer) to joint angles [23]. First, a closed-source sensor fusion algorithm was used to convert the three-sensor modulus to segment positions and orientations expressed in quaternions. Second, each quaternion was converted to joint angles according to ISB recommendations for standardization in the reporting of lower-limb kinematic data [26,27,28].

### 2.6. Data Analysis

The raw OMC-based and IMU-based joint angles were synchronized and filtered before the comparison between the two systems. The synchronisation was performed by matching the time of the peak knee flexion angle obtained from the two systems during the synchronisation task (i.e., a hard stomp with the right leg). Next, ten continuous strides were identified for each running trial with each stride cycle starting and ending with ipsilateral foot strike. The time instant of each ipsilateral foot strike was determined based on a built-in algorithm provided by Xsens MVN Analyze. The raw joint angles obtained from both systems were then filtered using a fourth-order, zero-lag, low-pass Butterworth filter with a cutoff frequency of 8 Hz for each stride cycle. A residual analysis was conducted to determine that 8 Hz was the optimal cutoff frequency for the present study [29].

### 2.7. Statistical Analysis

Multiple continuous and discrete metrics were employed to assess the validity of IMUs in measuring lower-limb joint angles and the pelvic orientation during high-speed running. The differences in continuous kinematics between the IMU and OMC systems were assessed using the root mean square error (RMSE), normalised RMSE (nRMSE), Pearson correlation coefficient (*r*), and statistical parametric mapping (SPM).

The RMSE between the OMC- and IMU-based joint angles was calculated for each one of the 1460 stride cycles. The nRMSE was computed by normalizing the RMSE with the average range of motion (ROM) for both systems [30]. The strength of Pearson correlation coefficient was interpreted as weak if *r* ≤ 0.35, moderate if 0.36 ≤ *r* ≤ 0.67, and strong if 0.68 ≤ *r* < 1 [31].

A one-dimensional SPM paired *t*-test was employed for each joint angle and running speed to identify specific regions of the stride cycle where significant differences in joint angles existed between the two systems. The significance level was set at *p* < 0.05. For each running speed, the SPM analysis was conducted individually based on the within-subject mean lower-limb joint angles generated for each participant per speed. The within-subject mean joint angle curves were averaged from two trials of 10 continuous stride cycles except in cases where only one trial was available. However, for the pelvic obliquity and rotation, the mean curve was based solely on the right-limb stride cycles due to asymmetries between the right- and left-limb stride cycles. All SPM analyses were performed using the open-source spm1D software library (version 3.0; www.spm1d.org) [32]. The D’Agostino–Pearson K2 test was conducted to check the normality of data distribution [33]. A non-parametric SPM paired *t*-test was conducted accordingly if the input data were not normally distributed.

Bland–Altman analysis was conducted to assess the discrete kinematic differences in peak joint angles between the two systems [12]. Peak pelvis and lower-limb joint angles obtained from the IMU and OMC systems were identified for all 1460 stride cycles to account for repeated measures. Bland–Altman plots were generated separately for each running speed to evaluate the accuracy of the IMU system by determining the systematic bias and limits of agreement (LoA). The calculations for bias and LoA varied based on whether the data were normally distributed, as determined by a D’Agostino–Pearson K2 test [33]. For normally distributed data, bias was calculated as the mean difference in peak angles between the two systems and LoA as bias ± 1.96 standard deviations. For non-normally distributed data, bias was calculated as the median difference in peak angles and LoA as bias ± 1.45 interquartile ranges. All Bland–Altman analyses were performed using the open-source Bland–Altman and Correlation Plot toolbox [34].

To assess the impact of running speed on the agreement between the two systems, separate linear mixed models were constructed for lower-limb joint angles. Each model included the RMSE between the joint angles obtained from the OMC and IMU systems as the dependent variable. The fixed effect in the models was the peak speed measured for each trial, while participant identification was included as a random effect. The RMSE for each lower-limb joint angle was calculated for every trial using data from 10 continuous stride cycles. The fixed effects were considered significant at *p* < 0.05.

## 3. Results

### 3.1. Data Collection and Running Speeds

A total of 160 trials were initially collected from 20 participants at four distinct running speeds; however, 14 trials were excluded due to missing marker data. Specifically, there were 37, 36, 36, and 37 trials available at 70%, 80%, 90%, and 100% of the maximal running speed, respectively. Altogether, 1460 stride cycles were analysed from 1600 total possible stride cycles (10 cycles × 2 trials × 4 speeds × 20 participants). The peak running speeds averaged across participants were 5.36 ± 0.55 m/s (70%), 6.02 ± 0.60 m/s (80%), 6.66 ± 0.71 m/s (90%), and 7.09 ± 0.73 m/s (100%).

### 3.2. Continuous Measurements of the Lower-Limb Joints

The mean RMSE and nRMSE values were below 10° and 10%, respectively, for the hip, knee, and ankle joints across all running speeds except for the ankle joint at the maximal speed (Table 1). The hip joint exhibited the highest average RMSE values across all speeds, ranging from 6.8° to 7.8°, while the ankle joint displayed the highest average nRMSE values, ranging from 8.4% to 10.4%. The patterns of the IMU-based joint angles measured for all speeds were strongly correlated with the corresponding references, with the lowest mean *r* values of 0.96 for the ankle joint at the maximal speed.

**Figure 2:**
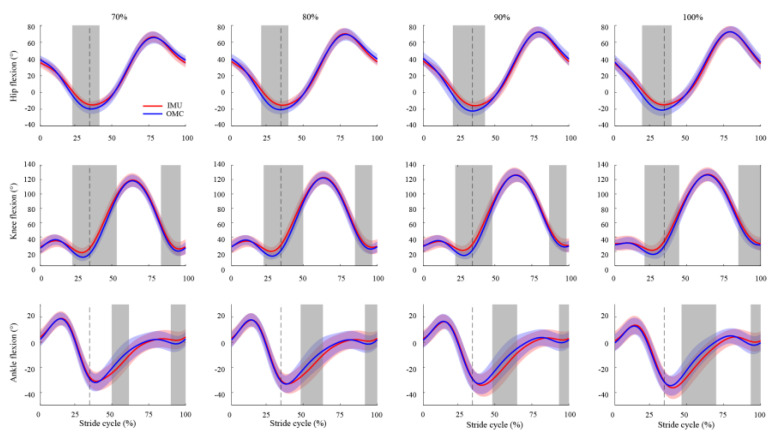
Comparison of the sagittal plane hip, knee, and ankle joint angles throughout one stride cycle, between IMU-based (red) and OMC-based (blue) measurements, at different running speeds (70%, 80%, 90%, and 100% of the maximal running speed). Positive angles represent the hip flexion, knee flexion, and ankle dorsiflexion. The shaded areas (red and blue) represent ± 1 standard deviation from the mean values. The dashed vertical line indicates the end of the stance phase for each running speed. The grey area highlights significant differences in joint angles between the IMU and OMC systems, as indicated by SPM analysis.

In comparison to the OMC system, the analysis of SPM revealed that the IMU system exhibited some notable distinctions (Figure 2). During ipsilateral toe-off (22–43% stride cycle for all speeds), the IMU system generated a significantly greater flexion in the hip and knee joints (*p* < 0.05), with a mean RMSE ranging from 6.2° to 8.1°. Similarly, the IMU-based knee flexion was also significantly higher (*p* < 0.05) during the terminal swing (82–100% stride cycle for all speeds), with a mean RMSE ranging from 6.5° to 7.6°. As for the ankle joint, the IMU system displayed significantly more plantarflexion during the first half of the swing phase (*p* < 0.01) and significantly less (*p* < 0.05) during the second half. Specifically, the mean RMSE for the ankle flexion during the first half of the swing phase was approximately twice as high (ranging from 5.0° to 6.0° across all speeds) as during the second half (ranging from 2.3° to 2.8° across all speeds).

### 3.3. Discrete Measurements of the Lower-Limb Joints

**Figure 3:**
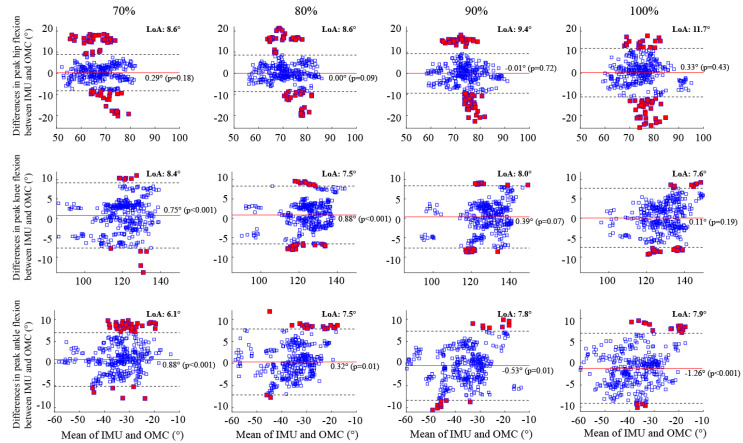
Bland–Altman plots illustrate the peak flexion angles of the hip, knee, and ankle during running at four different speeds. Each plot shows the average peak flexion angles on the horizontal axis and the differences between the IMU and OMC systems on the vertical axis. The horizontal red bar represents the systematic bias, while the black dotted lines indicate the upper and lower bounds of the limits of agreement. Data points outside the limits of agreement are represented by red squares. LoA = Limits of Agreement.

Bland–Altman analysis revealed that the systematic bias between the OMC and IMU systems was less than 1° for all lower-limb joint angles at each running speed except for the ankle joint at the maximal speed (Figure 3). Specifically, the peak ankle flexion angle exhibited a significant systematic bias ranging from −0.88° (*p* < 0.001) at 70% speed to 1.3° (*p* < 0.001) at the maximal speed. In contrast, a close-to-zero insignificant bias was observed in the peak hip flexion angles regardless of the running speed. For the peak knee flexion angle, significant biases of 0.75° (*p* < 0.001) and 0.88° (*p* < 0.001) were observed at 70% and 80% of the maximal running speed, respectively, while no significant bias was found at 90% and 100% of the maximal running speed.

Overall, the peak ankle flexions demonstrated tighter LoA (<8.0°) across all speeds compared to the peak hip flexions (<12°) and knee flexions (<9.0°) (Figure 3). No statistically significant bias was found in the peak hip flexions for all running speeds, while a significant bias of less than 1.5° was observed in the peak ankle flexions. A minimum of 83%, 92%, and 89% of the peak hip, knee, and ankle flexion measurements, respectively, fell within their respective LoA at all speeds.

**Figure 4:**
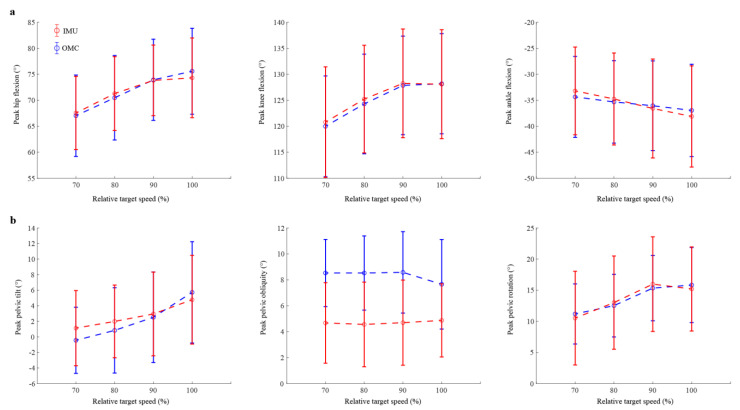
(**a**) The peak flexion angles of the hip, knee, and ankle and (**b**) the pelvic orientation (tilt, obliquity, and rotation) during running at four different speeds. Results are presented as mean (open circles) plus and minus one standard deviation (vertical error bars) of the peak values obtained for 18 participants (refer to Supplementary Materials Figures S1–S6 for individual results).

Averaging across all participants, both systems showed that the peak hip and knee flexion angles increased with running speed, while the peak ankle flexion angle exhibited a slight decrease as running speed increased (see Figure 4a). The standard deviations obtained from both systems largely overlapped for all lower-limb joint angles at each running speed, with the difference between the mean peak angles derived from the IMU-based and OMC-based measurements being less than 1.0°. It is worth noting that individual variations were observed, with some participants demonstrating a closer alignment between the two systems compared to other participants (Supplementary Materials Figures S1–S3).

### 3.4. Continuous Measurements of the Pelvic Orientation

The mean RMSE values between the pelvic orientation obtained from OMC and IMU systems were in the range of 4.3° to 7.8° across all speeds (Table 2). Notably, the RMSE for the pelvic rotation (6.5° to 7.8° across all speeds) was consistently greater than that of the pelvic tilt (4.3° to 4.6° across all speeds) and obliquity (4.3° to 4.7° across all speeds). When considering the range of motion, the mean nRMSE for the pelvic tilt was consistently 1 to 2 times higher than that of the pelvic obliquity and pelvic rotation for each running speed. The highest nRMSE values for the pelvic tilt, obliquity, and rotation were 58.5%, 30.9%. and 39.6%, respectively. Furthermore, the Pearson correlation analysis revealed that, compared to the pelvic obliquity and rotation (with *r* ranging from 0.67 to 0.89 across all speeds), the IMU-based pelvic tilt demonstrated a weaker correlation with the OMC-based measurement (with *r* ranging from 0.44 to 0.55 across all speeds).

**Figure 5:**
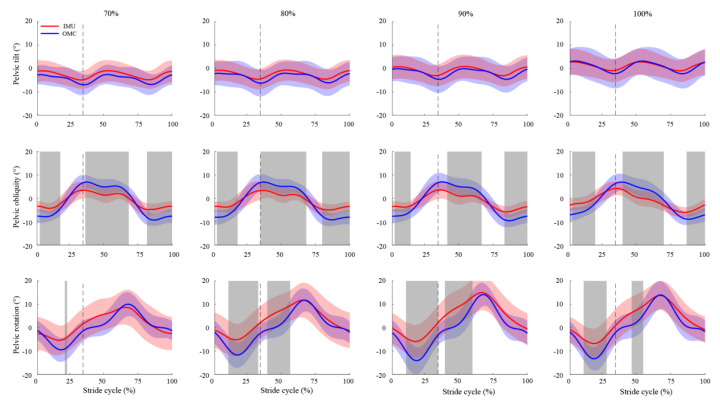
Comparison of the pelvic orientation (tilt, obliquity, and rotation) throughout one stride cycle between IMU-based (red) and OMC-based (blue) measurements at different running speeds (70%, 80%, 90%, and 100% of the maximal running speed). Positive angles represent the pelvic retroversion, left pelvic obliquity, and internal rotation. The shaded areas (red and blue) represent ± 1 standard deviation from the mean values. The dashed vertical line indicates the end of the stance phase for each running speed. The grey area highlights significant differences in joint angles between the IMU and OMC systems, as indicated by SPM analysis.

The SPM analyses demonstrated that the difference in the pelvic tilt measured by the OMC and IMU systems was insignificant when running at all speeds (*p* > 0.05) (Figure 5). However, the pelvic obliquity differed significantly between the two systems throughout the early, middle, and late stride cycle at all speeds (*p* < 0.01), with the mean RMSE values ranging from 3.9° to 4.1°, 4.0° to 4.5°, and 3.9° to 4.6°, respectively. Significant differences in the pelvic rotation were generally identified around the toe-off phase across all speeds except for 70% of the maximal running speed (*p* < 0.05).

### 3.5. Discrete Measurements of the Pelvic Orientation

**Figure 6:**
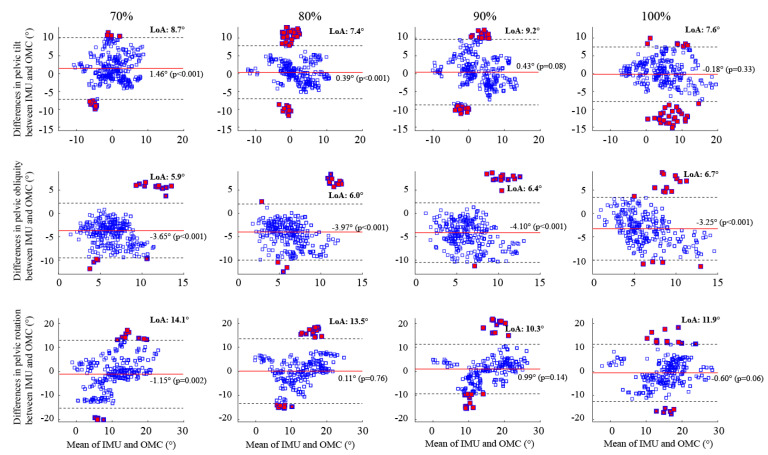
Bland–Altman plots illustrate the peak flexion angles of the pelvic orientation (tilt, obliquity, and rotation) during running at four different speeds. Each plot shows the average peak flexion angles on the horizontal axis and the differences between the IMU and OMC systems on the vertical axis. The horizontal red bar represents the systematic bias, while the black dotted lines indicate the upper and lower bounds of the limits of agreement. Data points outside the limits of agreement are represented by red squares. LoA = Limits of Agreement.

When examining all speeds, the IMU system consistently underestimated the peak pelvic obliquity compared to the OMC system, with a significant bias that varied between −3.3° at 100% (*p* < 0.001) and −4.1° at 90% (*p* < 0.001) of the maximal running speed (Figure 6). In contrast, the biases for the peak pelvic tilt and rotation were closer to zero, ranging from −0.18° to 1.5° and −1.1° to 0.99°, respectively, across all running speeds. Specifically, the IMU system overestimated the peak pelvic tilt, with significant biases of 1.5° (*p* < 0.001) and 0.39° (*p* < 0.001) at 70% and 80% of the maximal running speed, respectively. Furthermore, a significant bias of −1.1° (*p* = 0.002) in the peak pelvic rotation was observed only at 70% of maximal running.

Across all running speeds, the pelvic rotation exhibited both the highest RMSE and the widest LoA for its peak values when compared to the pelvic tilt and obliquity (Figure 6). Specifically, as the running speed increased from 70% to the maximal, the LoA for the peak pelvic rotation ranged from 11.9° to 14.1°, while the ranges were comparatively smaller for the pelvic tilt (7.4° to 9.2°) and obliquity (5.9° to 6.7°). At all speeds, a minimum of 89%, 93%, and 91% of the peak pelvic tilt, pelvic obliquity, and pelvic rotation measurements, respectively, fell within their respective LoA.

Both the OMC and IMU systems recorded an increase in the peak pelvic tilt and rotation as the running speed increased, while a similar trend was not evident in the peak pelvic obliquity (Figure 4b; see also Supplementary Materials Figures S4–S6 for individual results). In line with the systematic bias depicted in the Bland–Altman results (Figure 6), the IMU-based measurement of the peak pelvic obliquity consistently yielded lower values compared to the OMC-based measurements. The average discrepancy in the peak pelvic obliquity between the two systems ranged from 2.8° to 4.0°.

### 3.6. Effects of Running Speed on IMU Accuracy

The linear mixed model analysis revealed that running speed has a significant impact on the RMSE of the hip flexion (*p* < 0.001) and ankle flexion (*p* < 0.001) (Table 3). The estimated fixed-effect coefficients further indicated that a 1 m/s increase in speed corresponded to a 0.55° and 0.46° increase in the RMSE for hip and ankle flexions, respectively. The running speed had no significant effect on the RMSE of the knee flexion.

## 4. Discussion

The present study aimed to investigate the accuracy of IMUs in determining lower-limb sagittal plane kinematics and the pelvic orientation. We hypothesized that the RMSE between the OMC and IMU systems would be below 10° during high-speed running and that the running speed would affect the accuracy of IMU-based joint angles. Our results showed that the average RMSE values for hip, knee, and ankle joint angles were below 10° at all running speeds except for the ankle joint at the maximal speed (RMSE = 10.4°). We also observed statistically significant effects of the running speed on the hip flexion and ankle flexion but no significant impact on the RMSE values of the knee flexion, indicating its relative stability across different running speeds. Thus, our first hypothesis was supported, while the second hypothesis was partially supported, providing valuable insights into the accuracy of IMUs and their relationship with a high running speed.

Our findings on the RMSE between the IMU-based and OMC-based lower-limb joint angles align with results of previous studies. Wilmes et al. [35] have shown that RMSEs were less than 10° for the sagittal plane hip and knee flexions when comparing the IMU system with the OMC system at the maximal sprint speed (6.6 ± 0.3 m/s). Dorschky et al. [36] also found that the RMSEs for hip, knee, and ankle joint angles were below 10°, concomitant with a strong correlation (*r* > 0.9) between the IMU and OMC when subjects ran at speeds ranging from 3.0 m/s to 5.0 m/s. Consistent with our findings, Dorschky et al. [36] reported a decreasing trend in RMSE values as the joint moved from the hip (RMSE = 8.7 ± 3.2°), to the knee (5.3 ± 3.0°), and finally to the ankle (4.6 ± 1.7°). A similar proximal-to-distal decrease in the RMSE was also observed in Wilmes et al. [35], with the sagittal plane hip joint angle showing an RMSE approximately 1.5° greater than the knee joint angle.

Additionally, we noted strong validity in the measurement of lower-limb joint angles by IMUs, as indicated by both low nRMSE values (<10%) and a strong correlation (*r* > 0.9) across stride cycles for each running speed. It is important to highlight that a low RMSE does not necessarily imply a low nRMSE. This distinction can be exemplified by our findings, where the ankle joint exhibited the lowest RMSE and the largest nRMSE compared to the hip and knee joints (Table 1). This discrepancy can be primarily attributed to the ankle joint’s smaller range of motion relative to the other two joints (Figure 2).

Our SPM results indicated that the IMU system overestimated the hip and knee flexion angles around the ipsilateral toe-off region (Figure 2). Specifically, the IMU-based hip and knee flexion angles exhibited a slightly greater flexion during a 22–38% and 22–43% stride cycle, respectively, across all running speeds. These regions corresponded to the peak extensions for both the hip and knee joints. Upon further examination, the mean RMSE values for the peak hip and knee extensions ranged from 7.7° to 9.3° and 7.1° to 8.4°, respectively, as the running speed increased from 70% to the maximal speed. Comparing our findings to previous studies reporting RMSE values between 5° and 10° during walking and moderate-speed running [6,10,37], our results support the utilization of IMUs for measuring hip and knee angles in the sagittal plane during sprinting. However, caution should be exercised when interpreting measurements obtained during the ipsilateral toe-off region.

Consistent with the SPM results, the Bland–Altman analysis demonstrated a good between-system agreement in the sagittal plane peak joint angles across all running speeds, with negligible biases and less than 10° LoA, except for the peak hip flexion at the maximal running speed (LoA = 11.5°) (Figure 3). While no significant bias was detected at any running speed, the peak hip flexion displayed the highest number of outliers (i.e., points falling outside the LoA) compared to the peak knee and ankle flexions. Out of 1460 stride cycles, 238 (16%), 89 (6%), and 97 (7%) exhibited outliers for the peak hip, knee, and ankle flexion angles, respectively. More than 92% of the 238 hip outliers across all running speeds were linked to four participants, with the remaining outliers attributed to two other participants. Therefore, the Bland–Altman analysis suggests that the present IMU could be a suitable alternative to the established OMC system for detecting peak lower-limb joint angles in the sagittal plane, although individual variations in performance among subjects should be considered.

One factor contributing to the hip outliers (i.e., red squares in the top panel of Figure 3) is the differences in the pelvic tilt between the two systems. The hip flexion angle, calculated as the sagittal plane angle between the thigh and pelvis, can be increased by directly flexing the thigh or by rotating the pelvis more posteriorly while maintaining the global orientation of the thigh. For the majority of the hip outliers beyond the LoA, we found that the IMU system tended to produce a more posterior pelvic tilt of approximately 10° at the peak hip flexion. Conversely, for over 50% of the hip outliers falling below the LoA, the IMU system tended to yield a more anterior pelvic tilt of approximately 10°. These findings highlight the significance of the pelvic tilt in explaining the differences in hip flexion angles between the two systems.

The accuracy of the present IMU system in estimating the pelvic orientation during high-speed running was comparatively less satisfactory than its accuracy in estimating lower-limb joint angles. While the mean RMSE values for the pelvic orientation remained below the 10° threshold at all speeds, the corresponding mean nRMSE values consistently exceeded 20%. Notably, the pelvic tilt demonstrated a mean RMSE value of under 5° at all speeds, yet its mean nRMSE values surpassed 55% (Table 2). These elevated nRMSE values can be attributed to the small ROM for the pelvic tilt. Therefore, these findings suggest that the present 10° RMSE threshold is too conservative for assessing the accuracy of the IMU-based pelvic orientation during high-speed running.

Compared to the IMU-based pelvic orientation, the ROM observed in the OMC-based pelvic orientation was consistently larger across all speeds (Figure 5). Specifically, the OMC-based ROM for the pelvic tilt, obliquity, and rotation were approximately 1.2, 1.7, and 1.3 times greater than the respective IMU-based ROM across all speeds. By comparison, the OMC-based ROM for the lower-limb joint angles was 0.98 to 1.1 times greater than the IMU-based counterparts. These between-system differences in ROM for the pelvic orientation were also reflected in their Bland–Altman results. The LoA for the peak pelvic tilt, obliquity, and rotation could be up to 109.8%, 43.7%, and 71.4% of their respective ROM, while the maximum value for all peak lower-limb joint angles was only 14.8% for the peak ankle flexion. These findings suggest that caution should be exercised when interpreting the IMU-based pelvic orientation during high-speed running, particularly for the pelvic tilt.

It is noteworthy that no statistical difference was found in the SPM results for the pelvic tilt at all speeds (Figure 5). This observation may appear inconsistent considering the moderate *r* value and the wide LoA noted for the pelvic tilt. This discrepancy can be explained by the presence of considerable between-subject variations in both OMC and IMU measurements of the pelvic tilt. As evident from Figure 5, the standard deviations of the pelvic tilt calculated during movements were either similar to or exceeded the associated ROM, indicating significant between-subject variations. This pronounced between-subject variation has the potential to increase the risk of Type II errors and thereby hinder the accuracy of SPM results.

Running speed was found to have a significant effect on the RMSE of the hip and ankle flexion angles (Table 3). While it is expected that increasing segment velocity would compromise the accuracy of IMU-based estimations of lower-limb joint angles [38], our linear mixed model analysis revealed that the knee flexion angle remained unaffected, and there was only a modest increase of less than 1° in the RMSE for the hip and ankle flexion angles per 1 m/s increment in speed. These findings align with the LoA for the peak hip and ankle flexions in Figure 3, displaying a monotonous increase from 8.6° to 11.5° and 6.1° to 7.9°, respectively, as running speeds increased from 70% to the maximal speed.

One limitation of this study is the relatively small sample size. While several studies on IMU validation have used a similar sample size, increasing the sample size would enhance the statistical power, enabling the detection of smaller effect sizes with greater precision. Moreover, the generalizability of our findings may be constrained, as this study only compares a single IMU with one OMC system. It is also worth noting that, while OMC systems are traditionally regarded as a reference due to their relatively high accuracy compared to other systems [39], they are not immune to measurement errors (e.g., marker occlusions and soft-tissue artefact). Consequently, the RMSEs we computed reflect the observed discrepancies between the two systems rather than the deviation between the IMU system and the ‘actual’ movement. Furthermore, we used a curved non-motorised treadmill and asked participants to maintain submaximal running speeds based on visual feedback. While IMUs have the advantage of being portable, about 70% of studies analysing IMU-based running kinematics are conducted indoors [17,18]. Future research is encouraged to validate IMUs for estimating overground running biomechanics in outdoor conditions where speed control is not necessary.

## 5. Conclusions

In summary, our findings indicate that the present IMU system is a valid tool for accurately estimating lower-limb joint angles in the sagittal plane during sprinting. The obtained measurements were in good agreement with those derived from the benchmark OMC system across a range of running speeds (70% to 100% of the maximal speed). However, the performance of the IMU-based pelvic orientation was less satisfactory, possibly due to considerable between-subject variation. Analysing the data through a linear fixed model revealed that the running speed exerted a statistically significant yet minor influence on the accuracy of the IMU-based hip and ankle flexion angles, with an estimated RMSE < 1° resulting from a 1 m/s speed increase.

## Figures and Tables

**Table 1:** Mean ± standard deviations of the root mean square error (RMSE), normalised RMSE (nRMSE), and correlation coefficient (*r*) for sagittal plane hip, knee, and ankle joint angles between IMU- and OMC-based measurements at four different running speeds. The number of participants (N) at each running speed varies based on trial availability.

		Relative Target Speed			
Joint Angle	Variable	70% (N = 19)	80% (N = 19)	90% (N = 18)	100% (N = 19)
Hip flexion	RMSE (°)	6.76 ± 3.18	6.95 ± 3.33	7.31 ± 3.03	7.83 ± 3.43
	nRMSE (%)	7.72 ± 3.24	7.54 ± 3.34	7.64 ± 3.00	8.18 ± 3.50
	*r*	0.99 ± 0.01	0.99 ± 0.01	0.99 ± 0.01	0.99 ± 0.01
Knee flexion	RMSE (°)	6.20 ± 2.10	5.96 ± 1.82	6.30 ± 1.85	6.02 ± 1.68
	nRMSE (%)	5.69 ± 1.95	5.38 ± 1.65	5.56 ± 1.66	5.27 ± 1.51
	*r*	0.99 ± 0.01	0.99 ± 0.00	0.99 ± 0.00	0.99 ± 0.00
Ankle flexion	RMSE (°)	4.44 ± 1.89	4.64 ± 2.28	5.33 ± 2.33	5.33 ± 2.37
	nRMSE (%)	8.45 ± 3.87	8.45 ± 3.79	9.72 ± 4.09	10.37 ± 4.83
	*r*	0.97 ± 0.03	0.98 ± 0.02	0.97 ± 0.03	0.96 ± 0.03

**Table 2:** Mean ± standard deviations of the root mean square error (RMSE), normalised RMSE (nRMSE), and correlation coefficient (*r*) for the pelvic orientation (tilt, obliquity, and rotation) between IMU- and OMC-based measurements at four different running speeds. The number of participants (N) at each running speed varies based on trial availability.

		Relative Target Speed			
Joint Angle	Variable	70% (N = 19)	80% (N = 19)	90% (N = 18)	100% (N = 19)
Pelvic tilt	RMSE (°)	4.31 ± 2.50	4.32 ± 2.75	4.59 ± 2.51	4.55 ± 2.93
	nRMSE (%)	56.10 ± 33.95	55.15 ± 38.50	58.30 ± 39.96	55.97 ± 46.44
	*r*	0.55 ± 0.38	0.49 ± 0.43	0.44 ± 0.49	0.54 ± 0.38
Pelvic obliquity	RMSE (°)	4.29 ± 1.30	4.45 ± 1.50	4.75 ± 1.60	4.62 ± 1.38
	nRMSE (%)	28.43 ± 8.44	29.33 ± 10.39	30.86 ± 10.67	31.34 ± 10.88
	*r*	0.83 ± 0.26	0.81 ± 0.30	0.75 ± 0.33	0.67 ± 0.35
Pelvic rotation	RMSE (°)	6.84 ± 4.09	7.37 ± 4.37	7.77 ± 4.64	6.47 ± 2.93
	nRMSE (%)	39.46 ± 31.10	38.41 ± 37.77	34.16 ± 34.34	28.20 ± 23.97
	*r*	0.79 ± 0.33	0.80 ± 0.30	0.85 ± 0.25	0.89 ± 0.13

**Table 3:** Linear mixed model results for the root mean square error (RMSE) of the sagittal plane hip, knee, and ankle joint angles with the peak running speed within a trial as the fixed effect and participant identification as the random effect.

				95% Confidence Interval	
Model	Term	Estimate	*p*-Value	Lower	Upper
RMSE Hip	Intercept	3.63	<0.001	1.66	5.6
	Peak Speed	0.56	<0.001	0.32	0.79
RMSE Knee	Intercept	7.34	<0.001	5.27	8.98
	Peak Speed	−0.19	0.11	−0.42	0.04
RMSE Ankle	Intercept	1.85	0.01	0.42	3.27
	Peak Speed	0.46	<0.001	0.27	0.66

## Data Availability

Data are available on reasonable request due to restrictions, e.g., privacy or ethical.

## Supplementary Materials

The following supporting information can be downloaded at: https://www.mdpi.com/article/10.3390/s23239599/s1, Figure S1: Mean (open circles) plus and minus one standard deviation (vertical error bars) of the peak hip flexion angles for individual participant running at four different speeds. Results are generated from a minimum of 10 consecutive stride cycles per participant for IMU (red) and OMC (blue) systems. Two participants (P19 and P20) were excluded due to missing marker data for at least one running speed. Figure S2: Mean (open circles) plus and minus one standard deviation (vertical error bars) of the peak knee flexion angles for individual participant running at four different speeds. Results are based on a minimum of 10 consecutive stride cycles per participant for IMU (red) and OMC (blue) systems. Two participants (P19 and P20) were excluded due to missing marker data for at least one running speed. Figure S3: Mean (open circles) plus and minus one standard deviation (vertical error bars) of the peak ankle plantarflexion angles for individual participant running at four different speeds. Results are based on a minimum of 10 consecutive stride cycles per participant for IMU (red) and OMC (blue) systems. Two participants (P19 and P20) were excluded due to missing marker data for at least one running speed. Figure S4: Mean (open circles) plus and minus one standard deviation (vertical error bars) of the peak pelvic tilts for individual participant running at four different speeds. Results are based on a minimum of 10 consecutive stride cycles per participant for IMU (red) and OMC (blue) systems. Two participants (P19 and P20) were excluded due to missing marker data for at least one running speed. Figure S5: Mean (open circles) plus and minus one standard deviation (vertical error bars) of the peak pelvic obliquities for individual participant running at four different speeds. Results are based on a minimum of 5 right-limb stride cycles per participant for IMU (red) and OMC (blue) systems. Two participants (P19 and P20) were excluded due to missing marker data for at least one running speed. Figure S6: Mean (open circles) plus and minus one standard deviation (vertical error bars) of the peak pelvic rotations for individual participant running at four different speeds. Results are based on a minimum of 5 right-limb stride cycles per participant for IMU (red) and OMC (blue) systems. Two participants (P19 and P20) were excluded due to missing marker data for at least one running speed.
