# Supplementary figures and images for: Validity of Inertial Measurement Units to Measure Lower-Limb Kinematics and Pelvic Orientation at Submaximal and Maximal Effort Running Speeds
Source: Sensors (Basel). 2023 Dec 4;23(23):9599. doi: 10.3390/s23239599 (PMC10708829; doi:10.3390/s23239599)

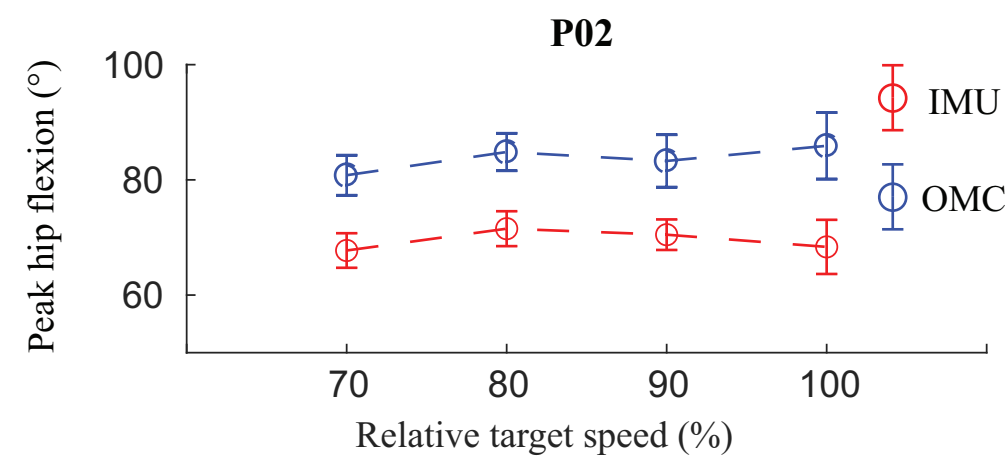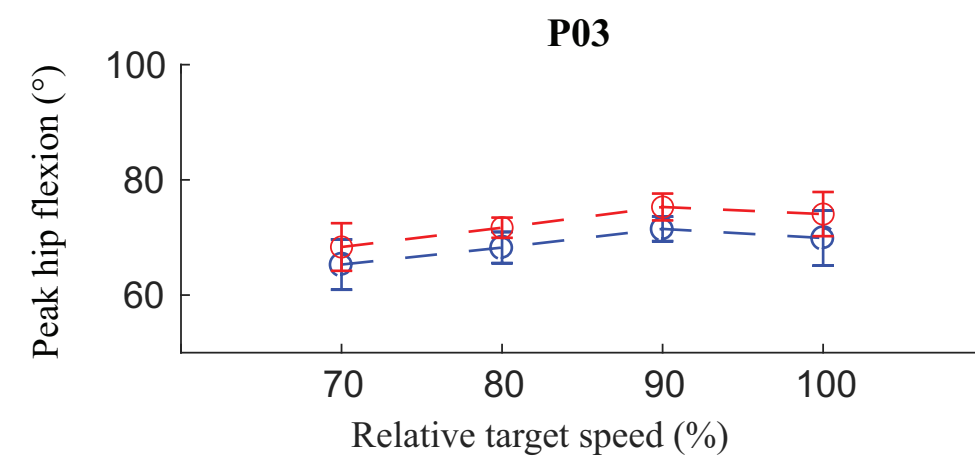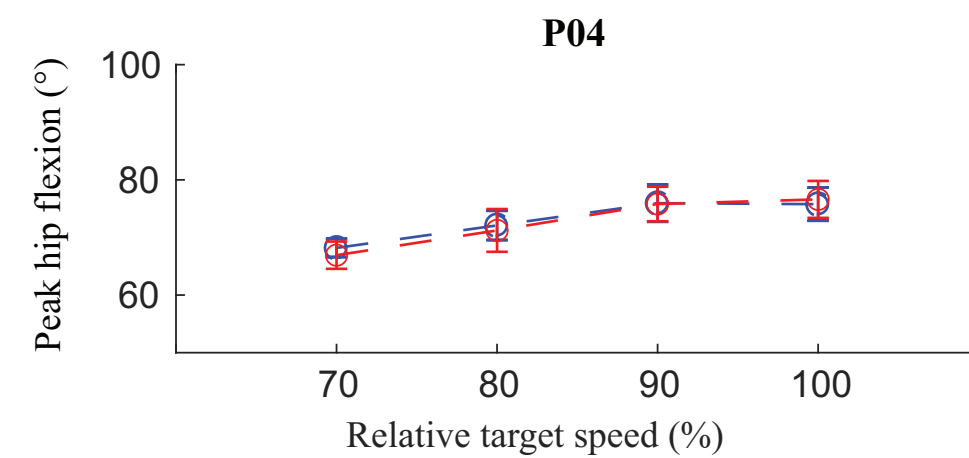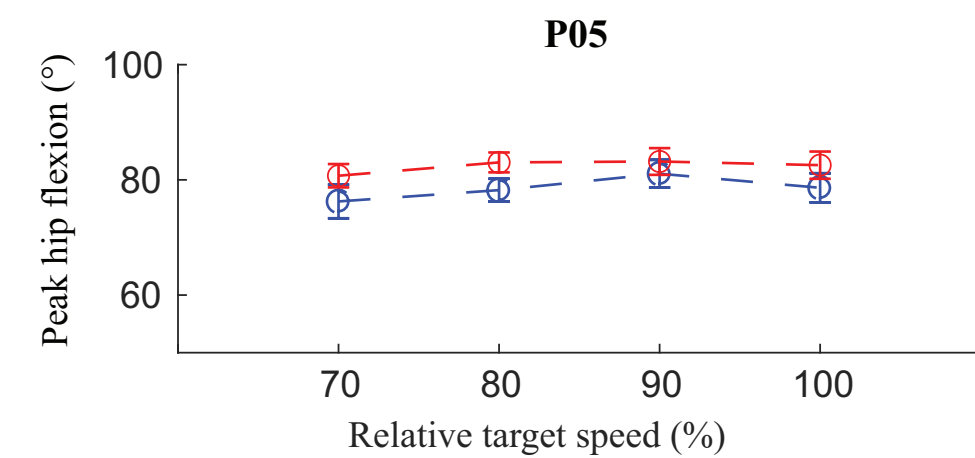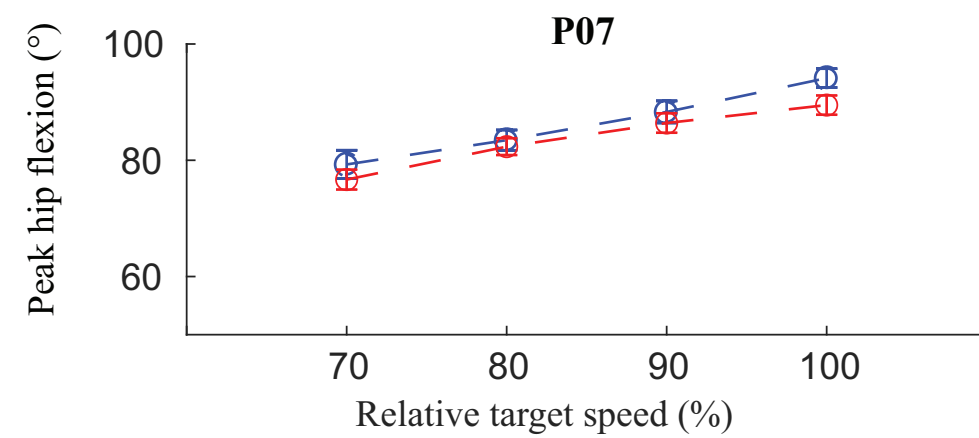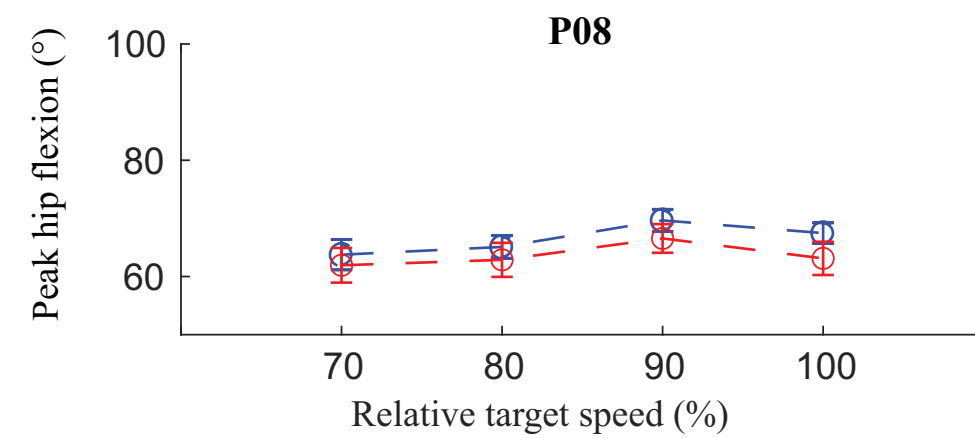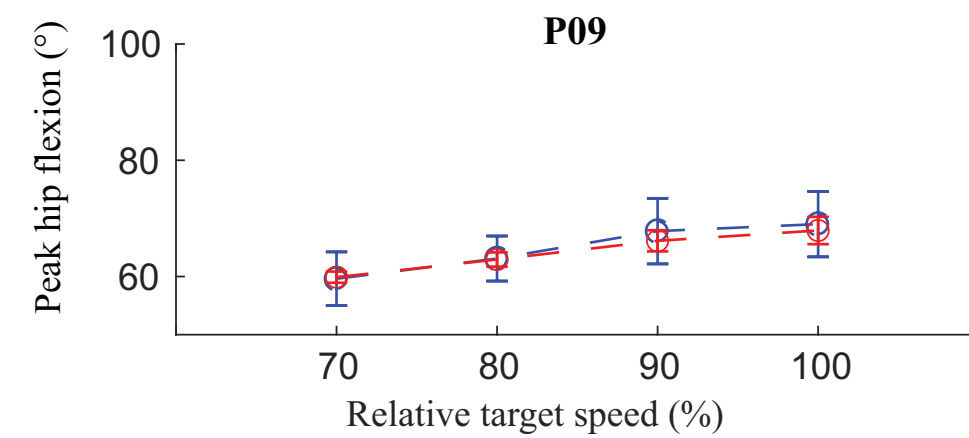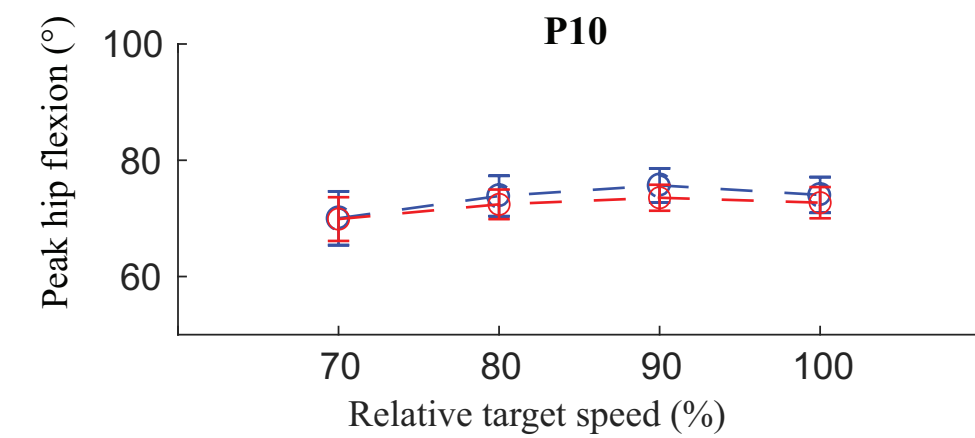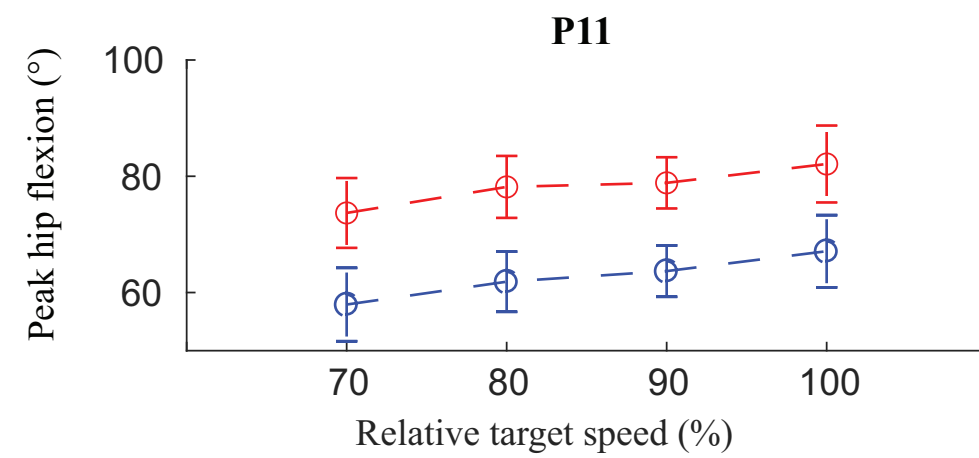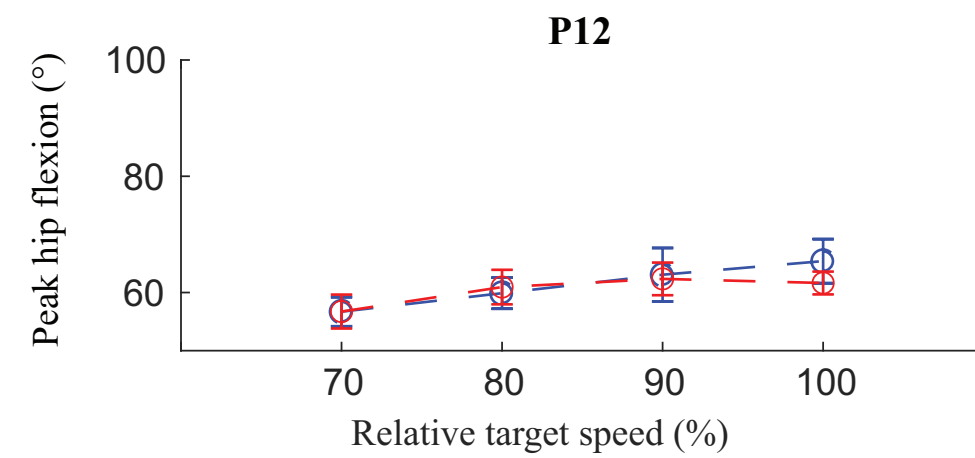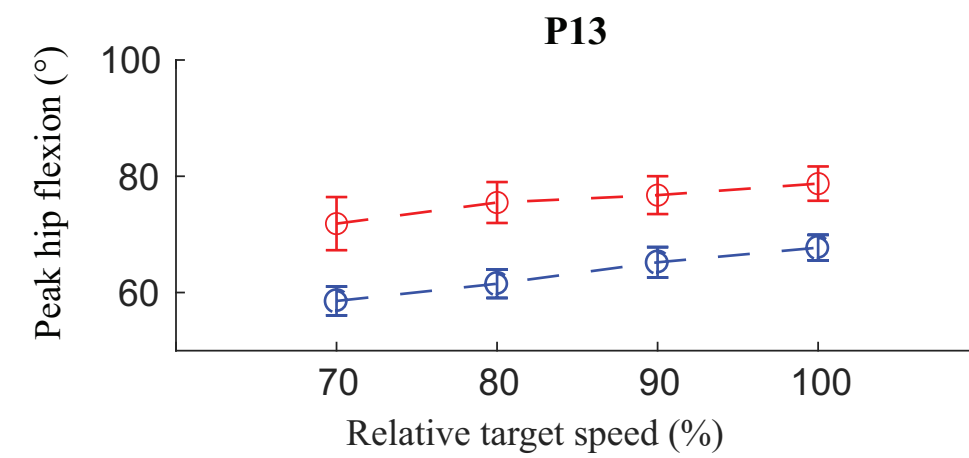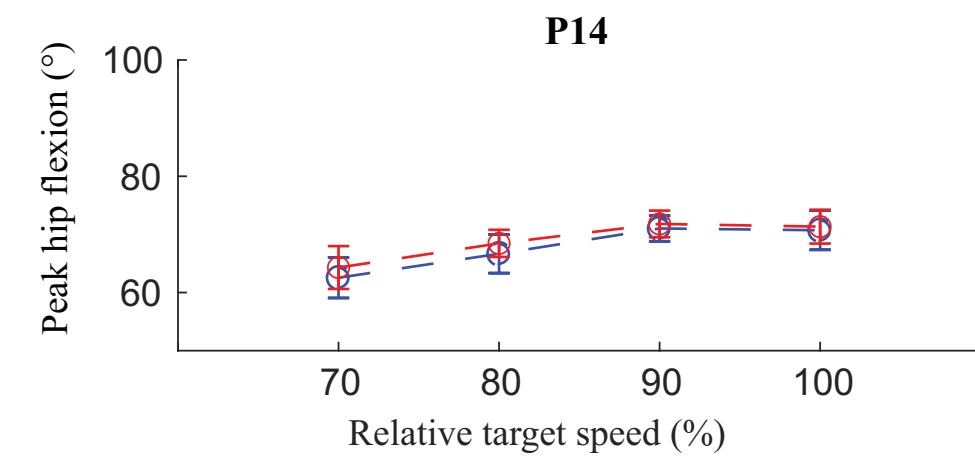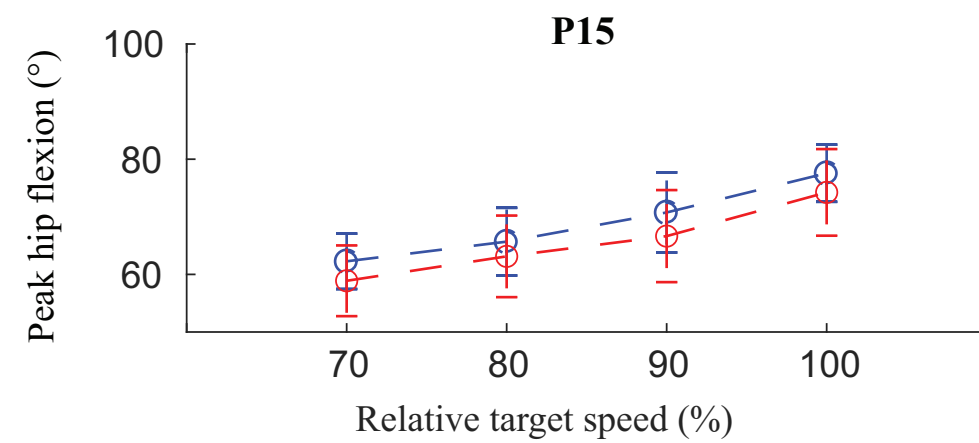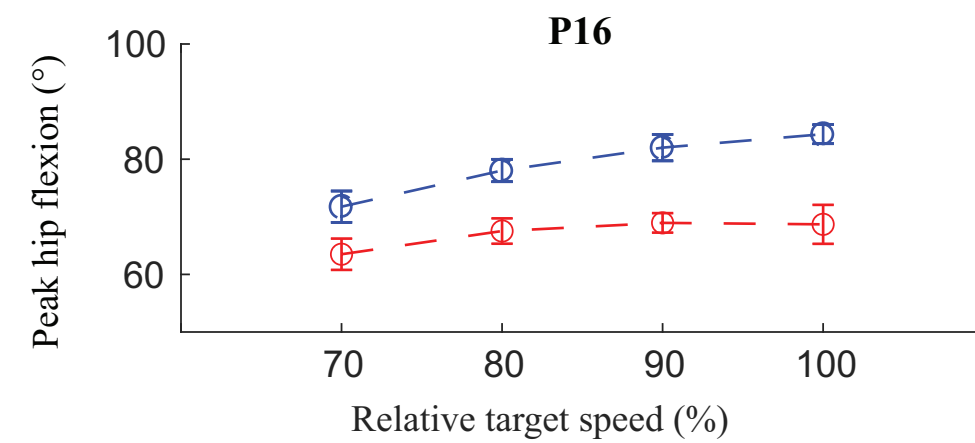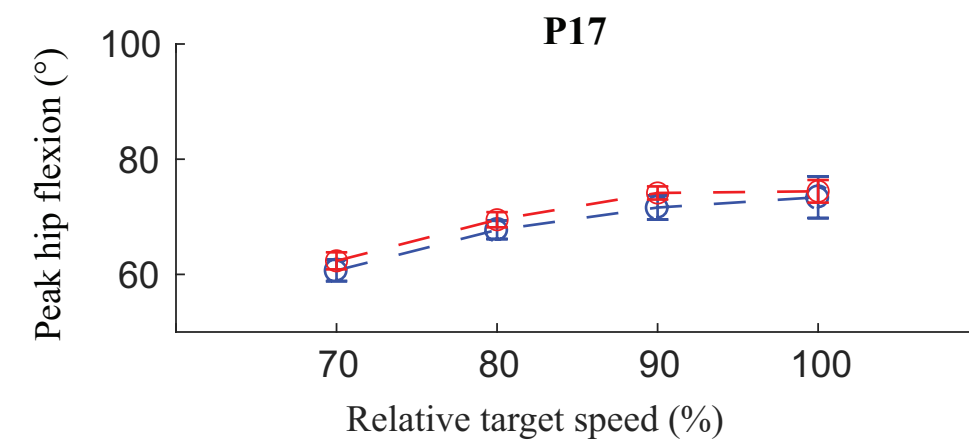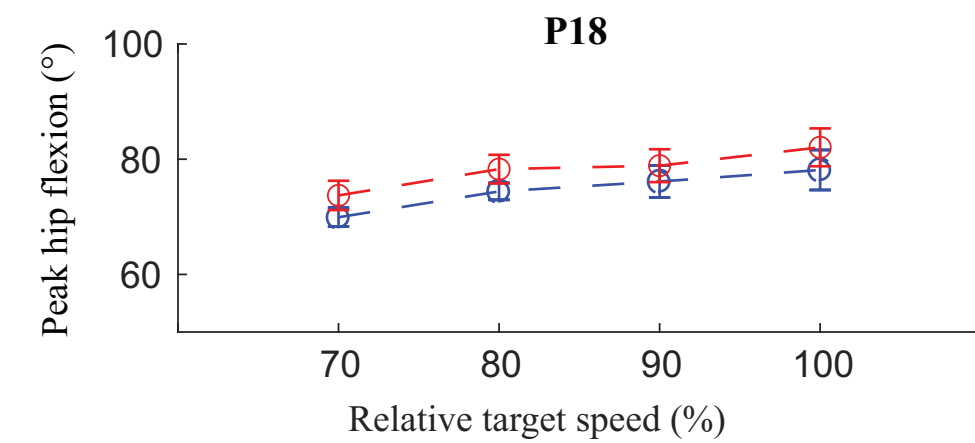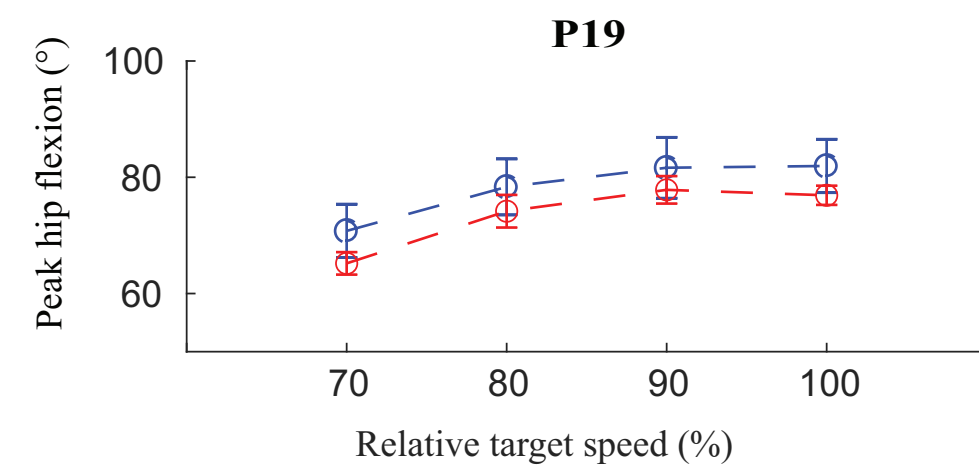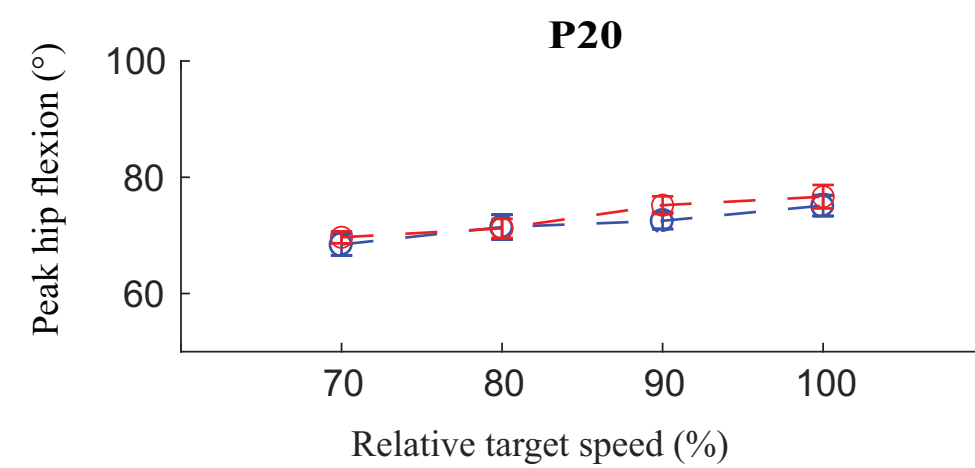

Supplement: Supplementary file 1 [file sensors-23-09599-s001.zip › FigS1_PeakHipFlexTrend.pdf]

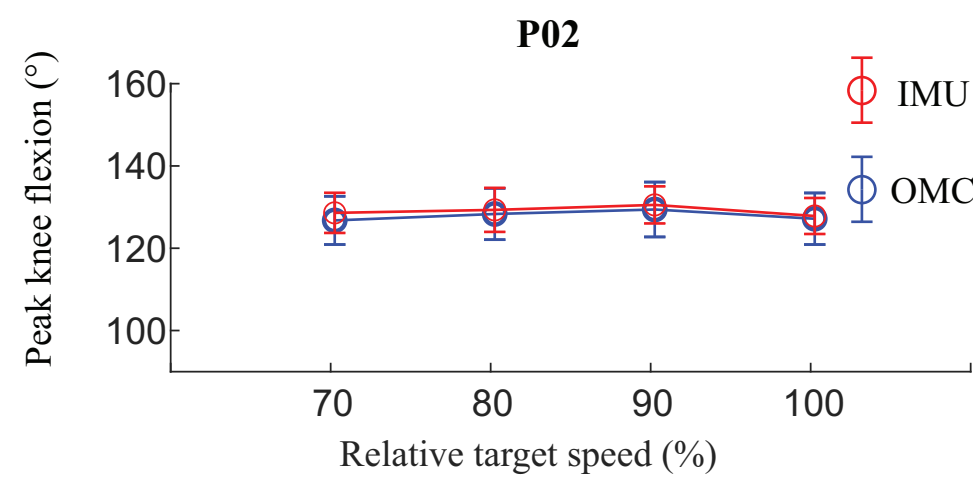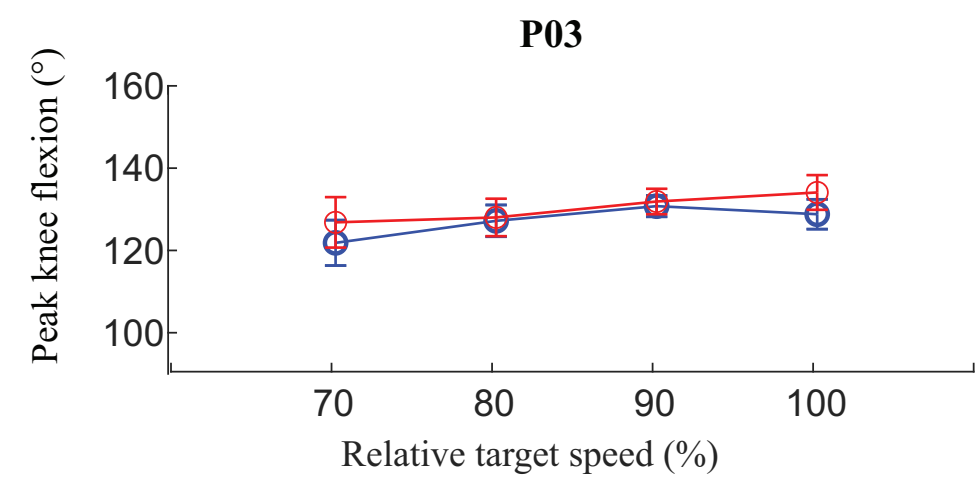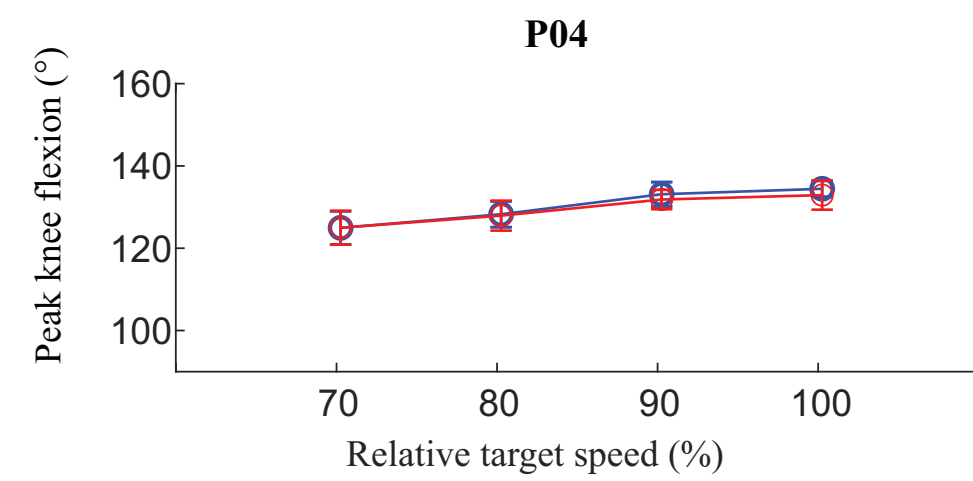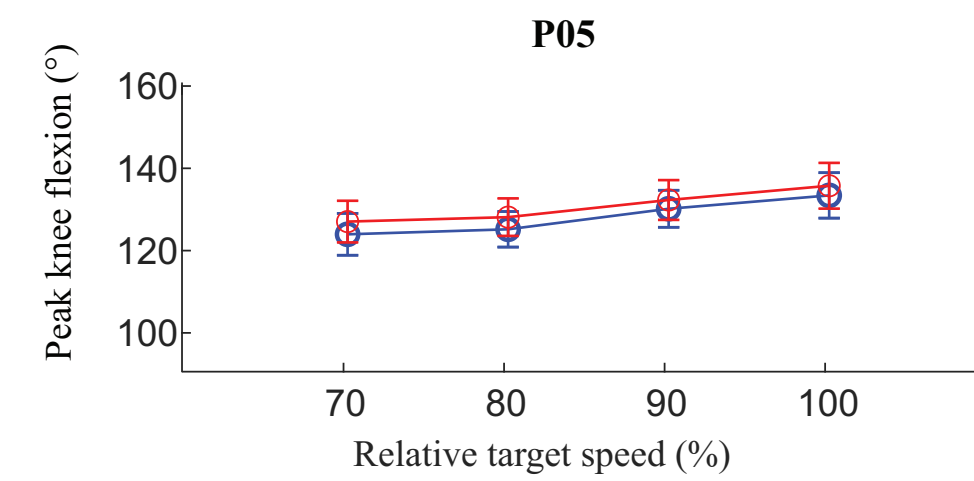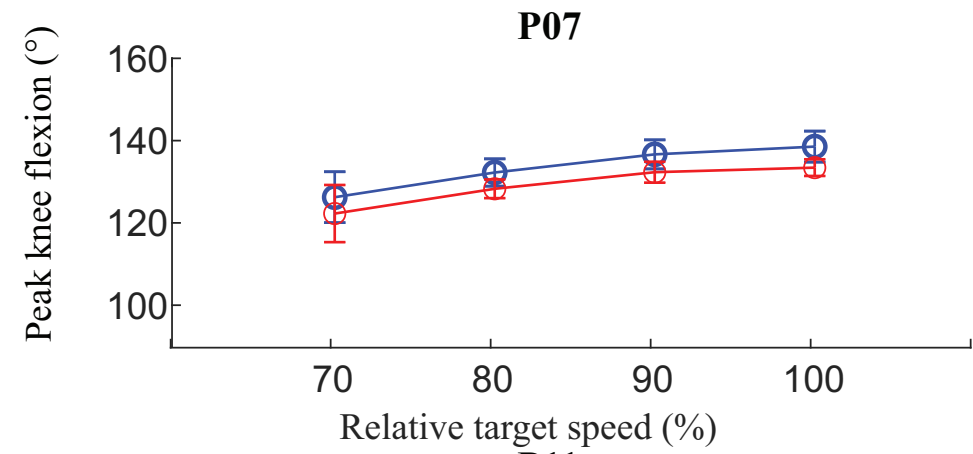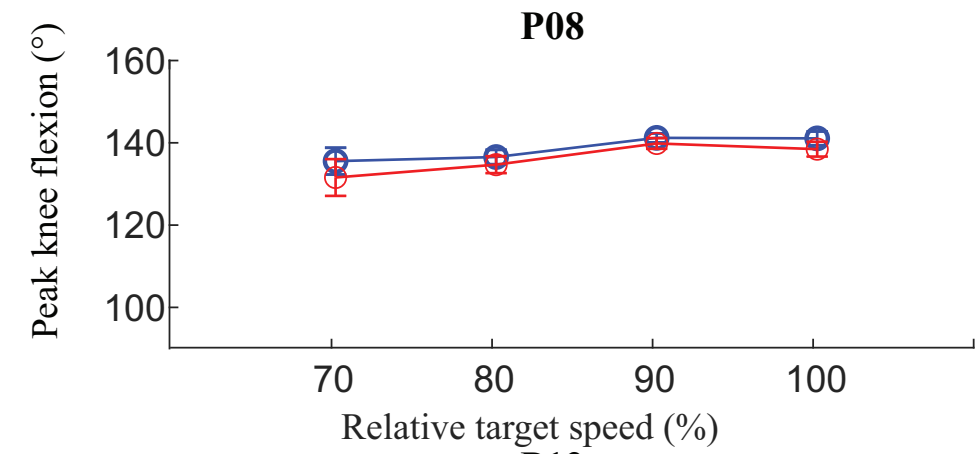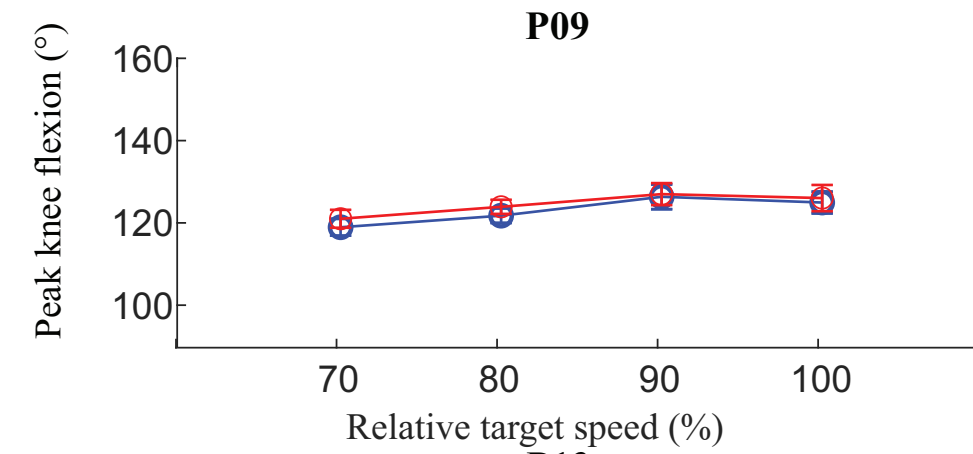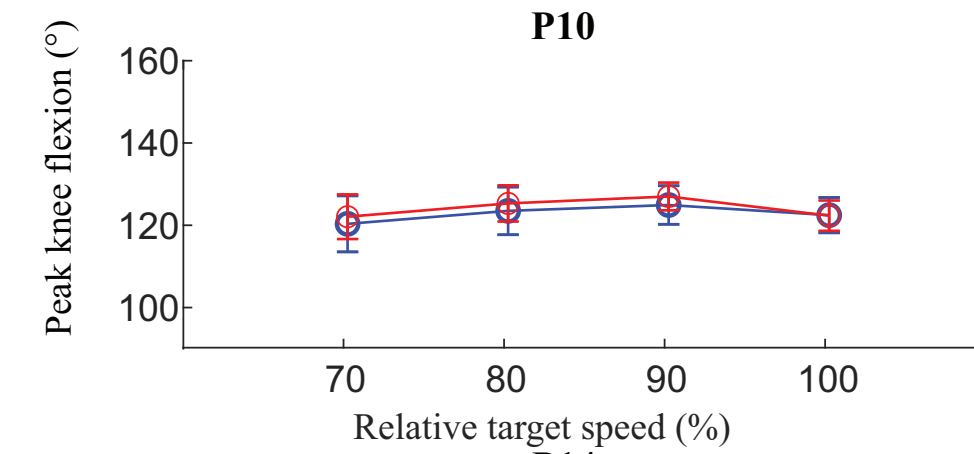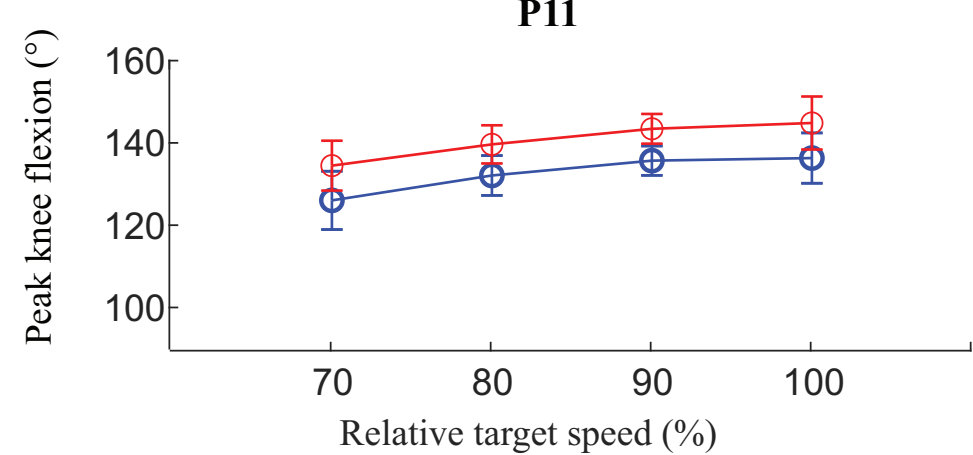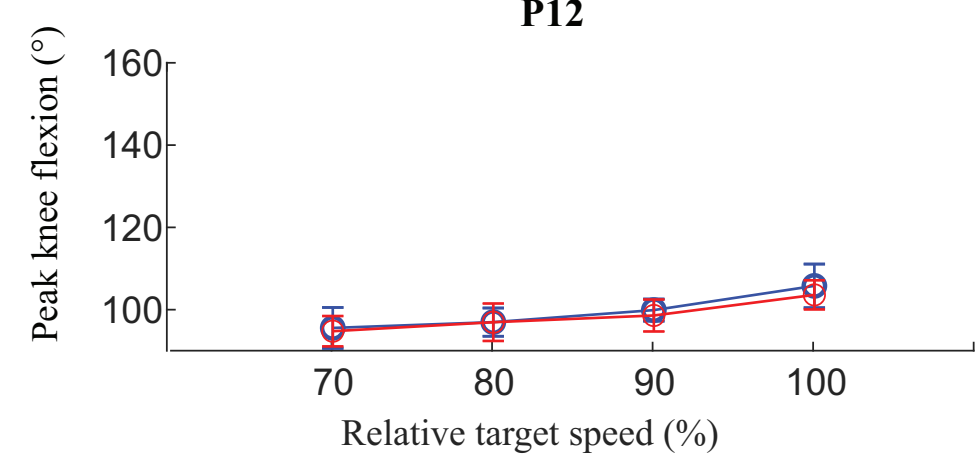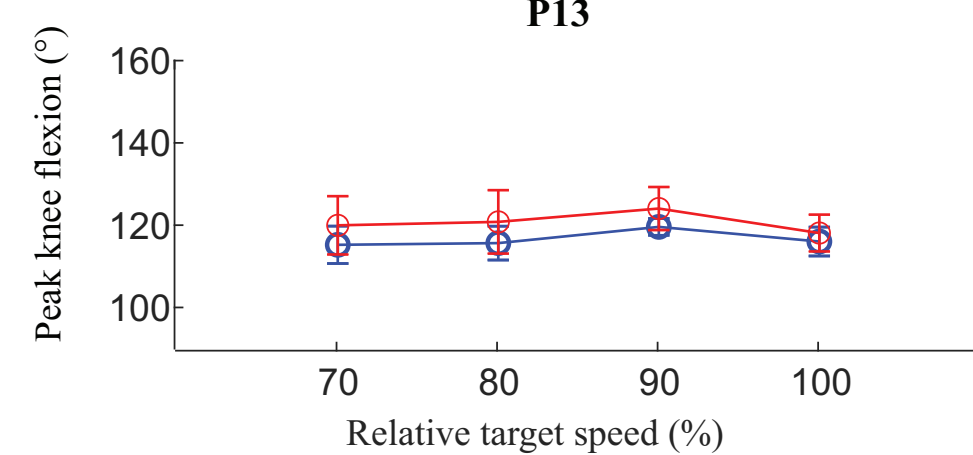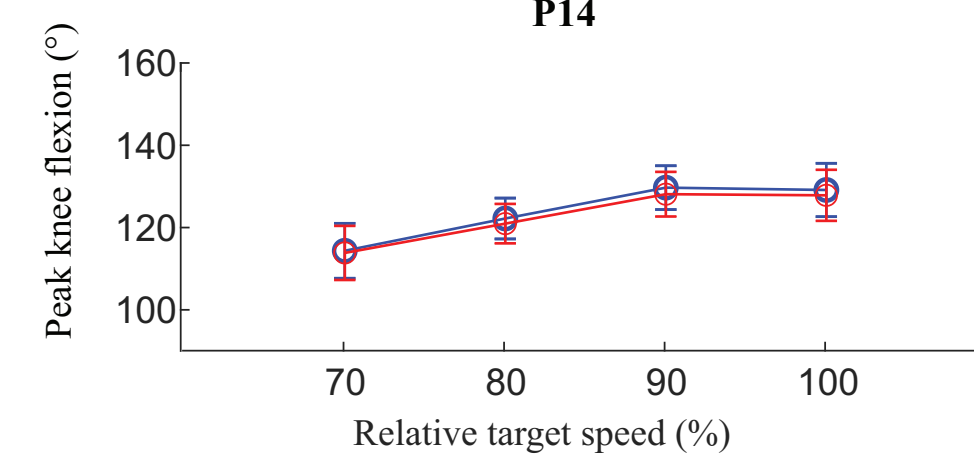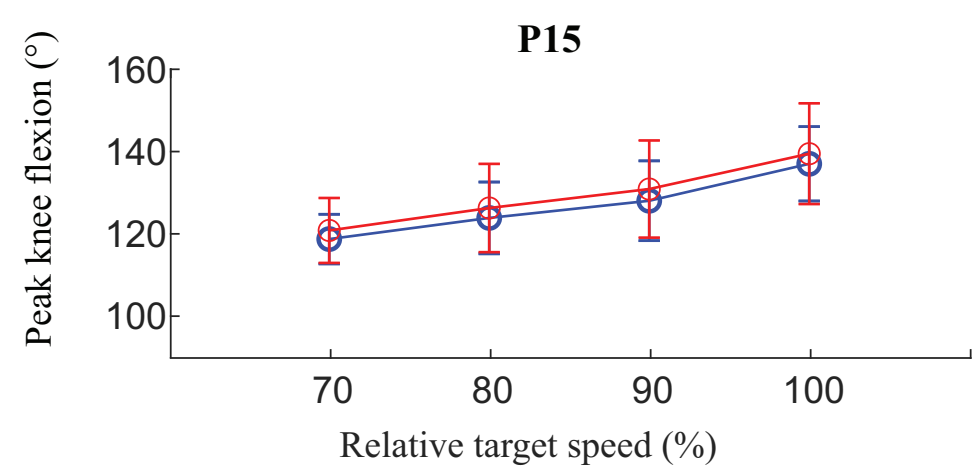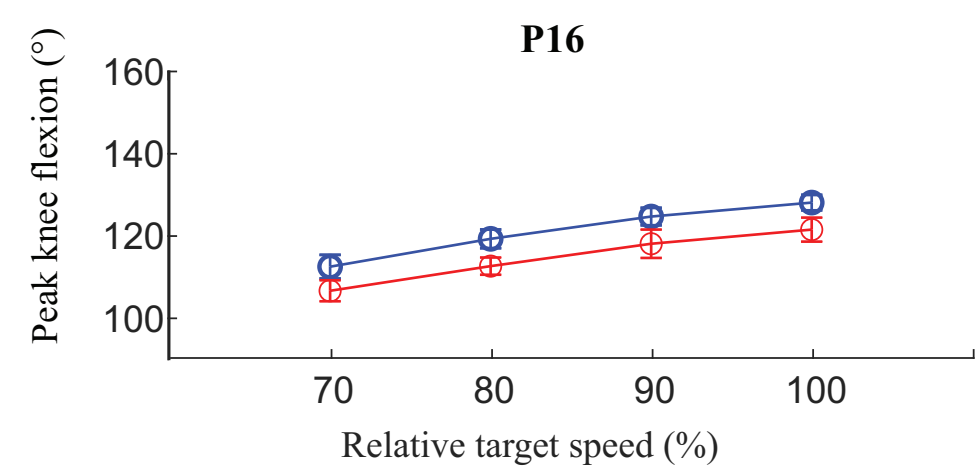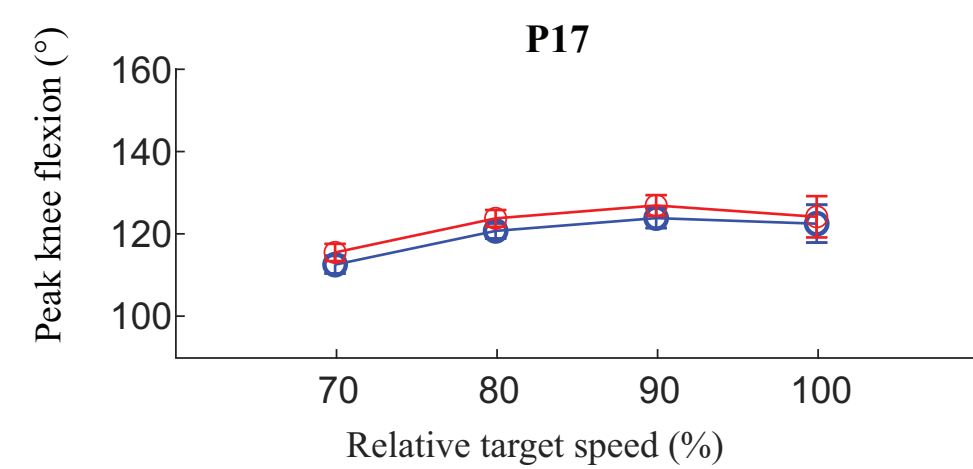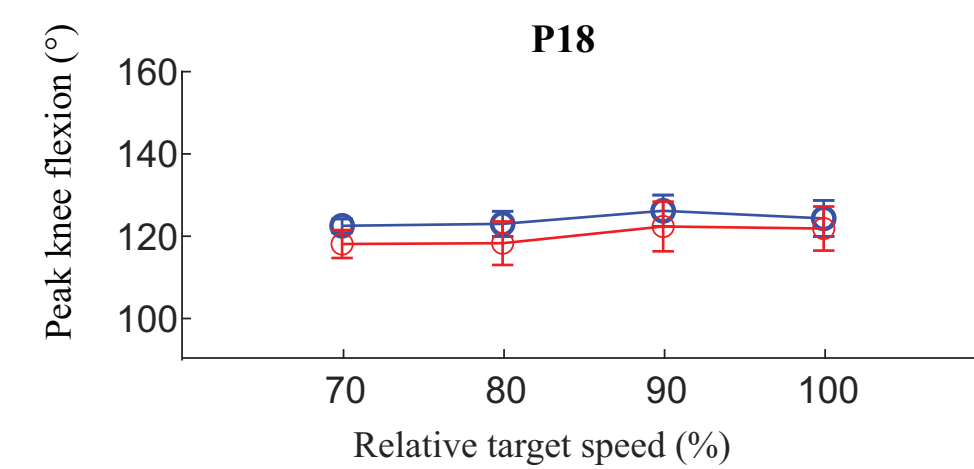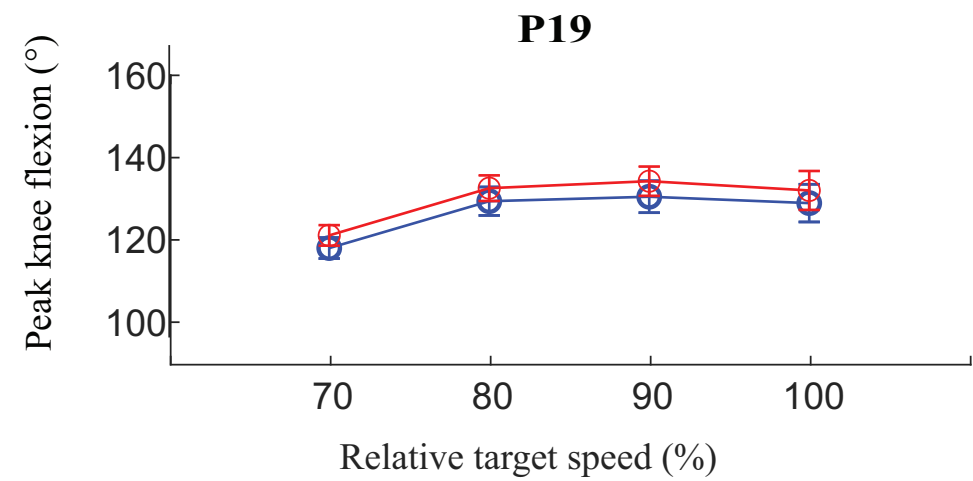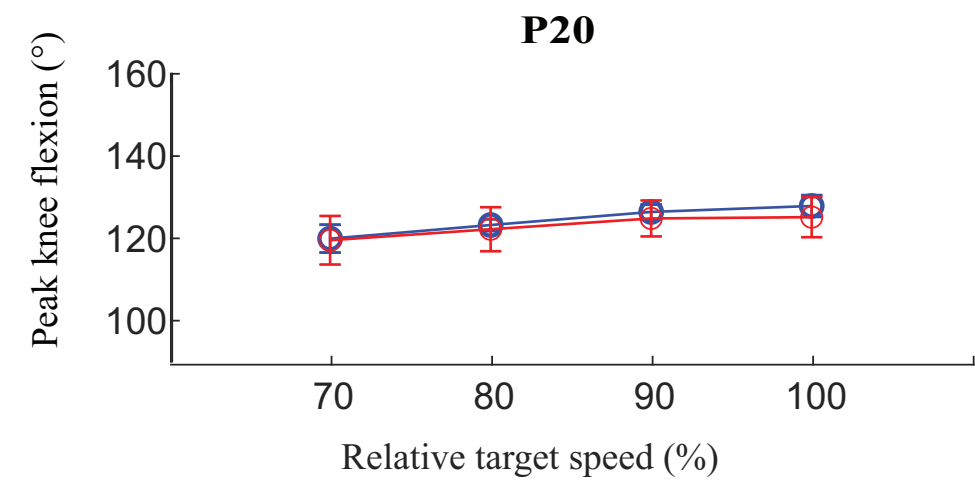

Supplement: Supplementary file 1 [file sensors-23-09599-s001.zip › FigS2_PeakKneeFlexTrend.pdf]

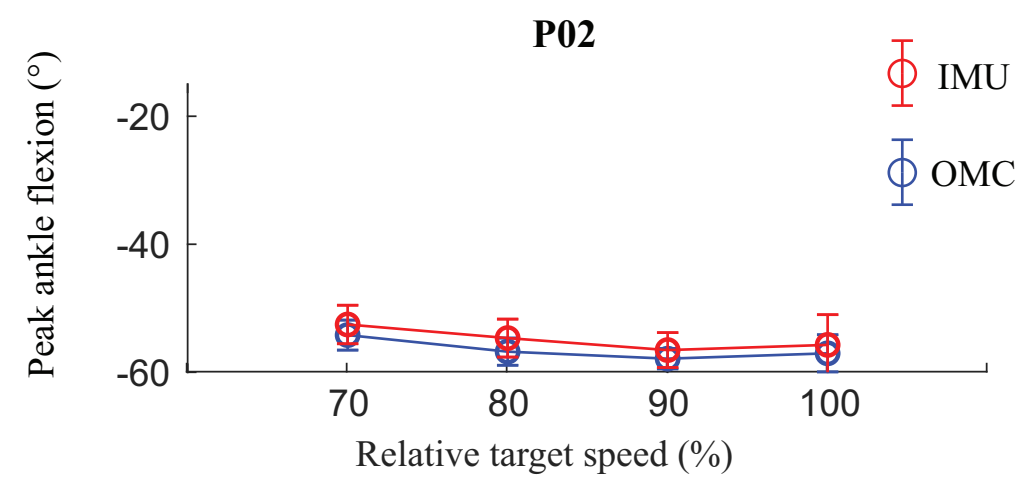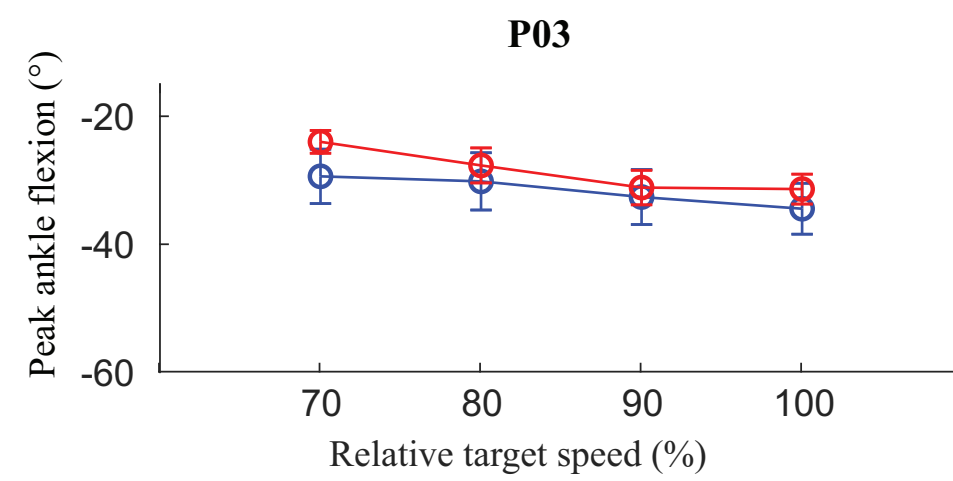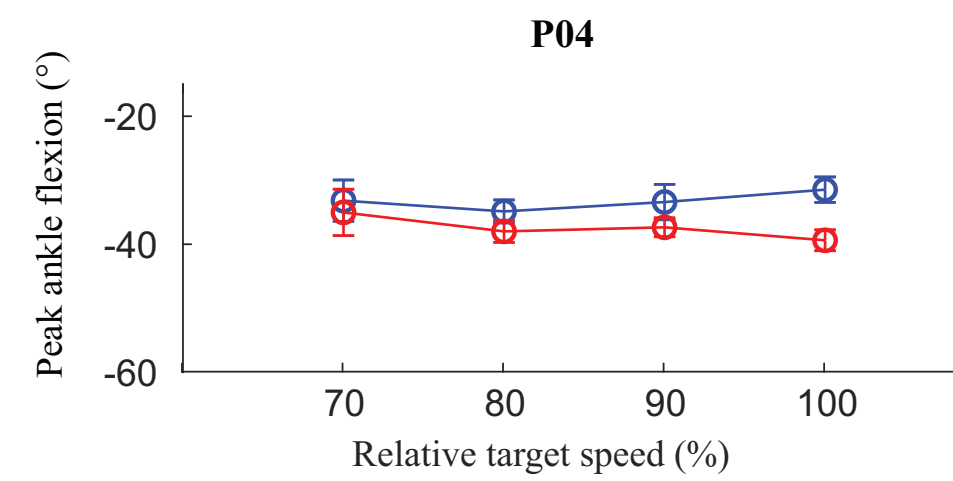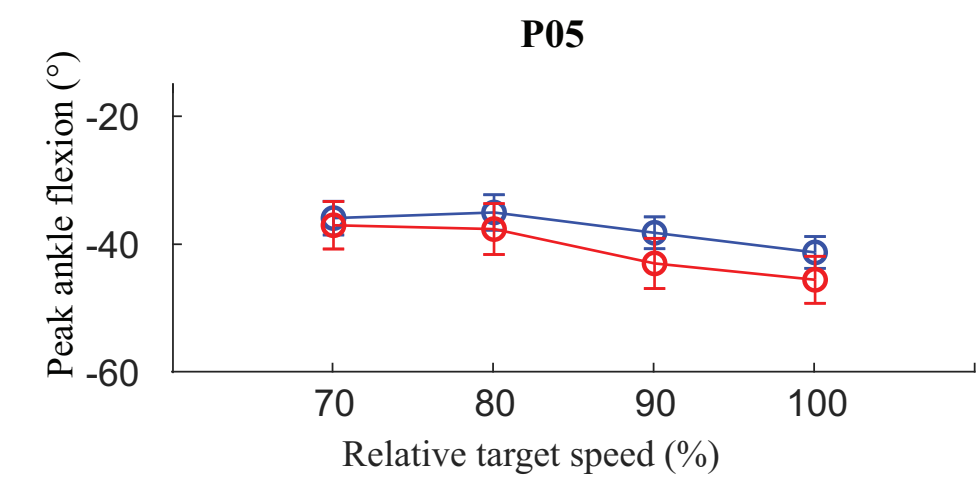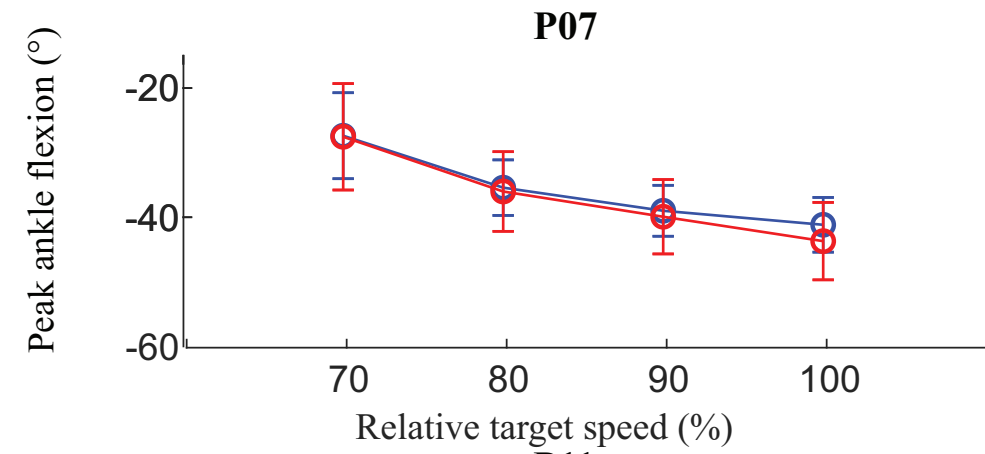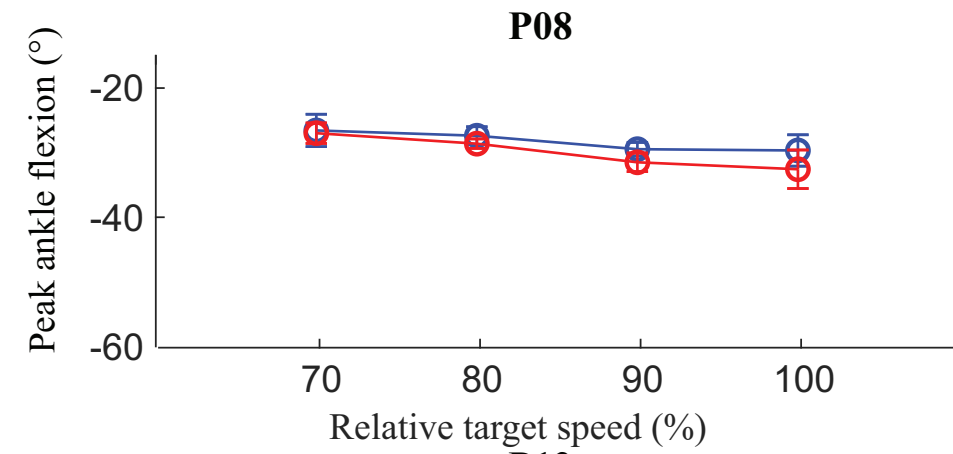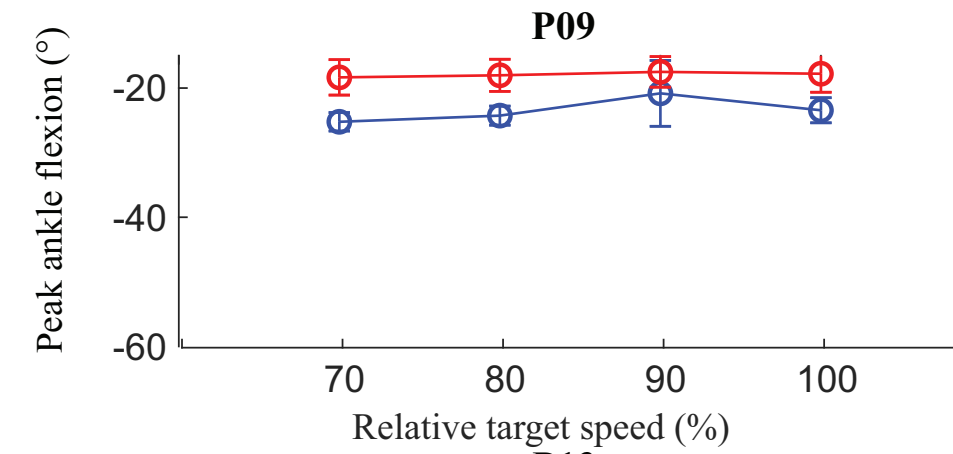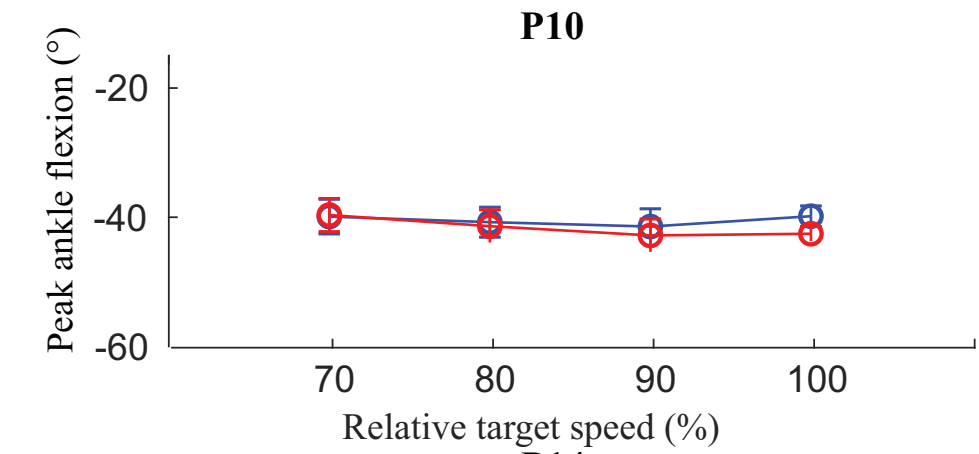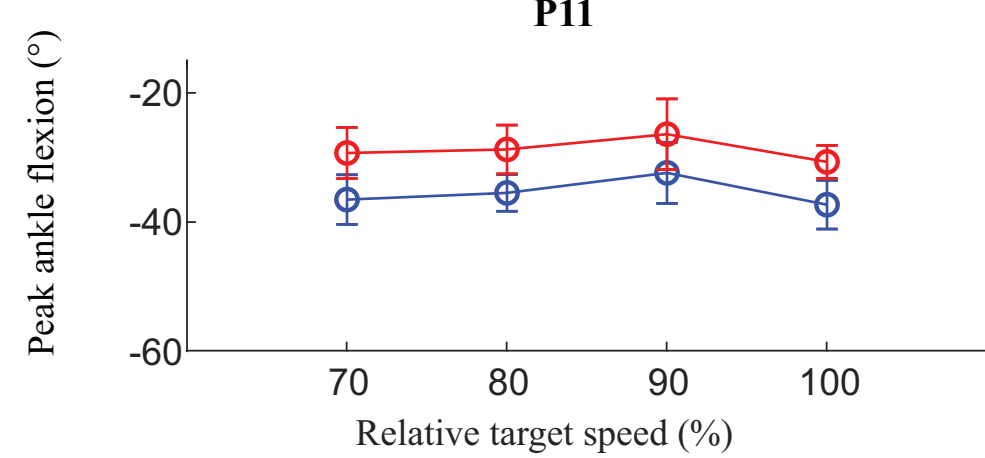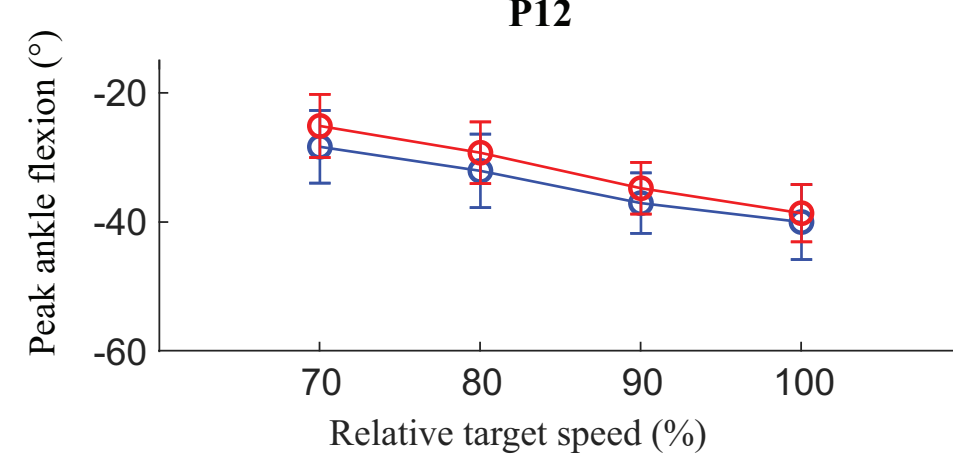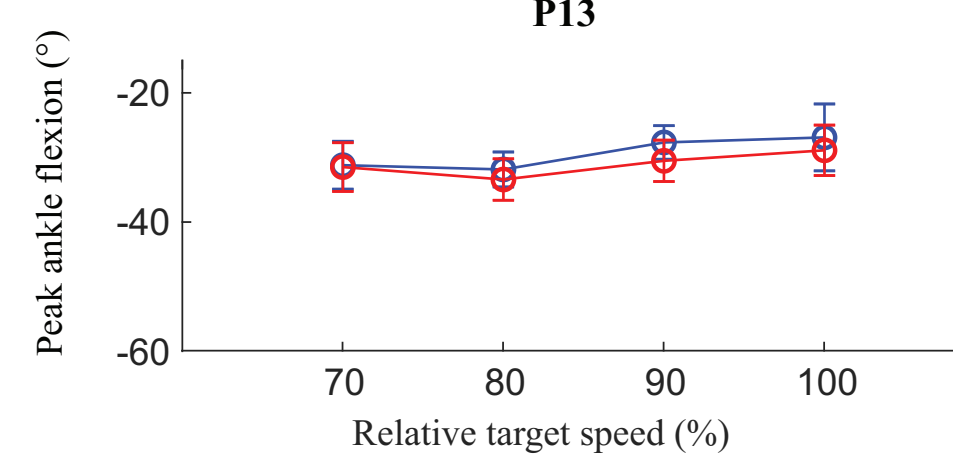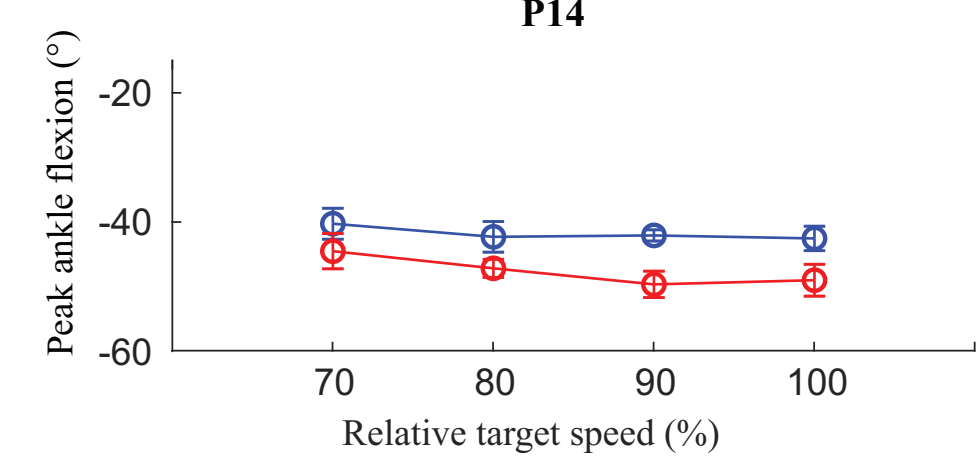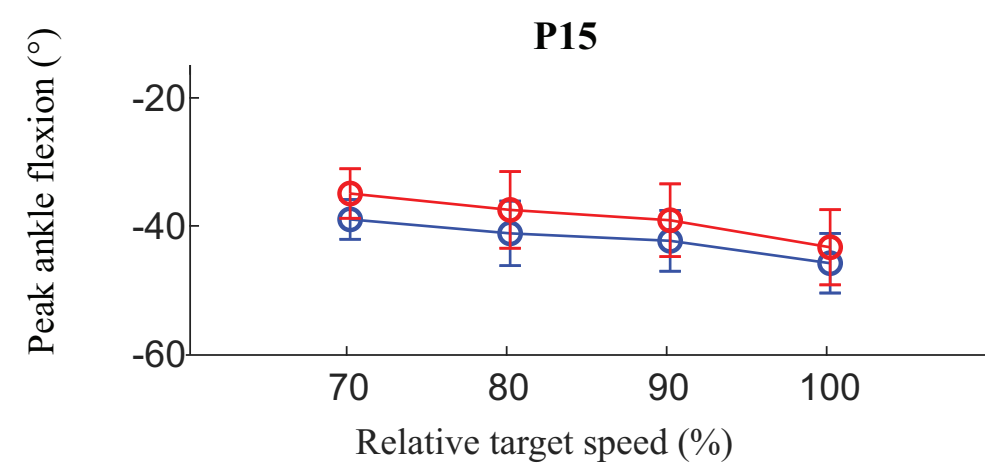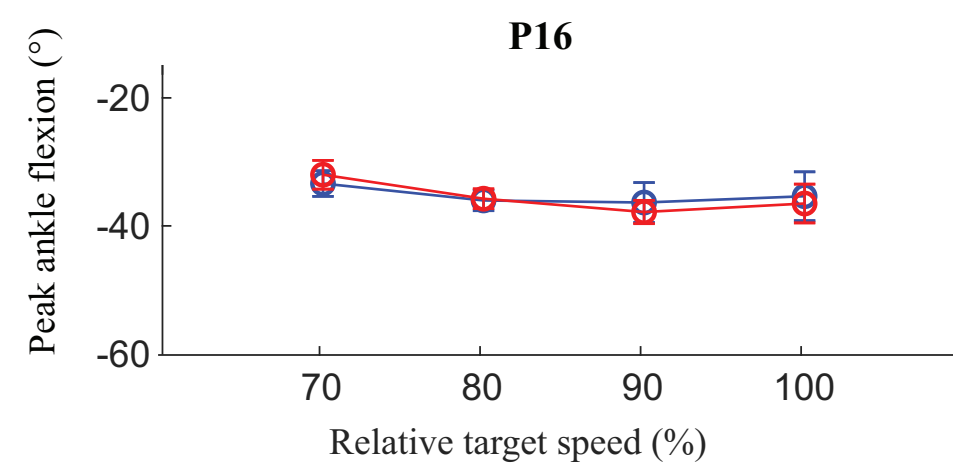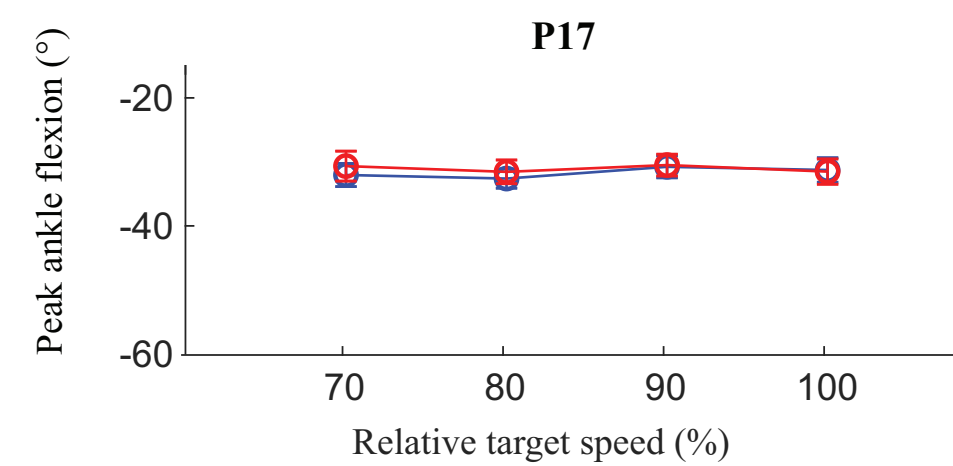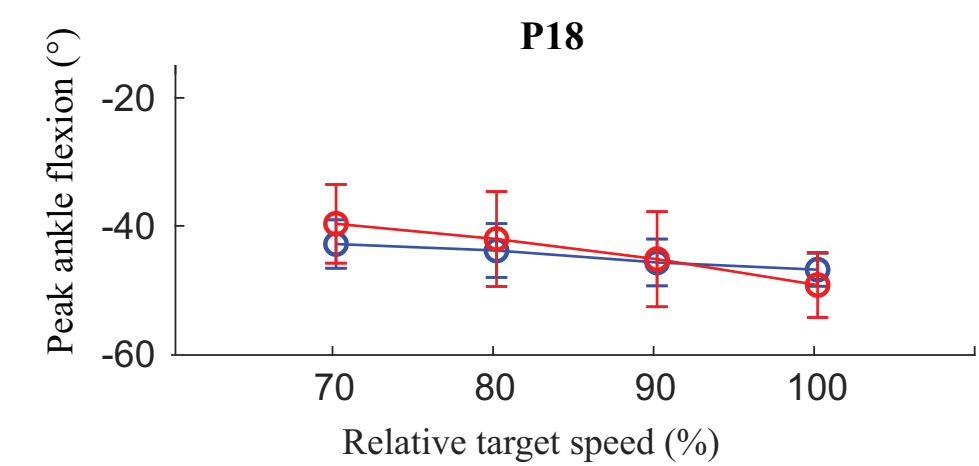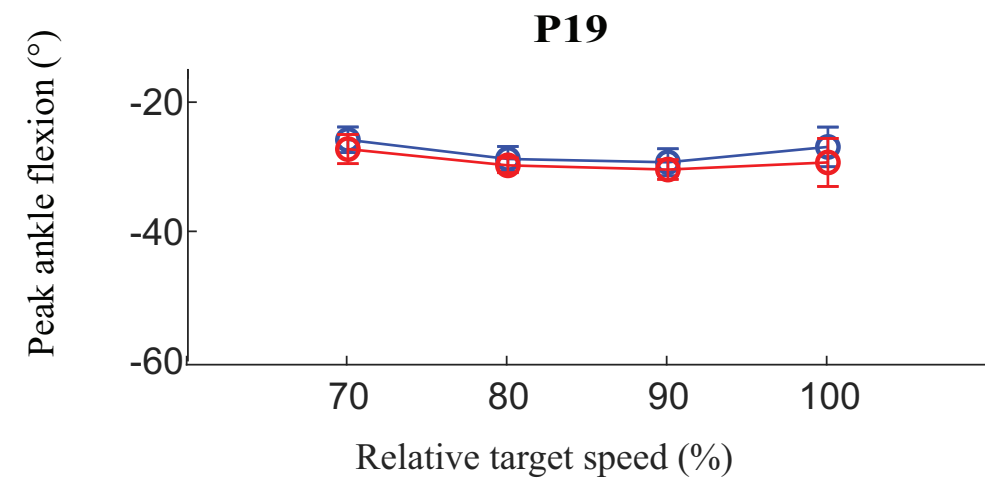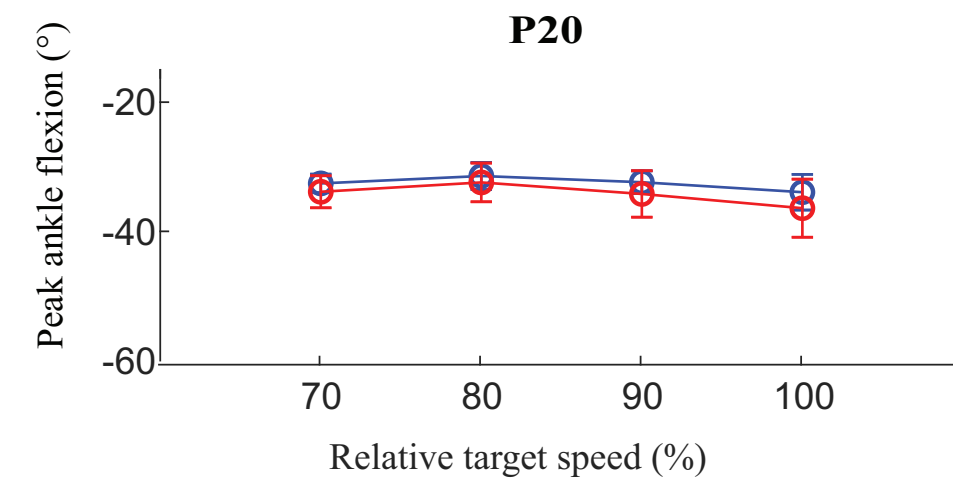

Supplement: Supplementary file 1 [file sensors-23-09599-s001.zip › FigS3_PeakAnkleFlexTrend.pdf]

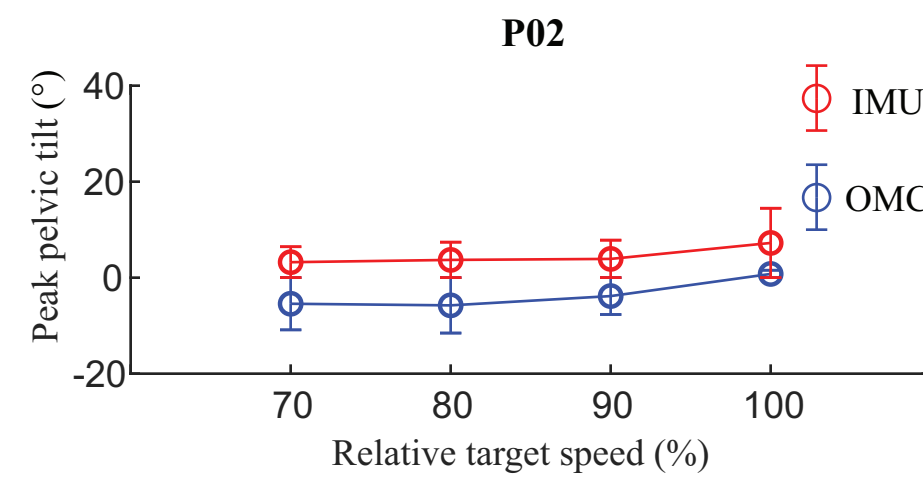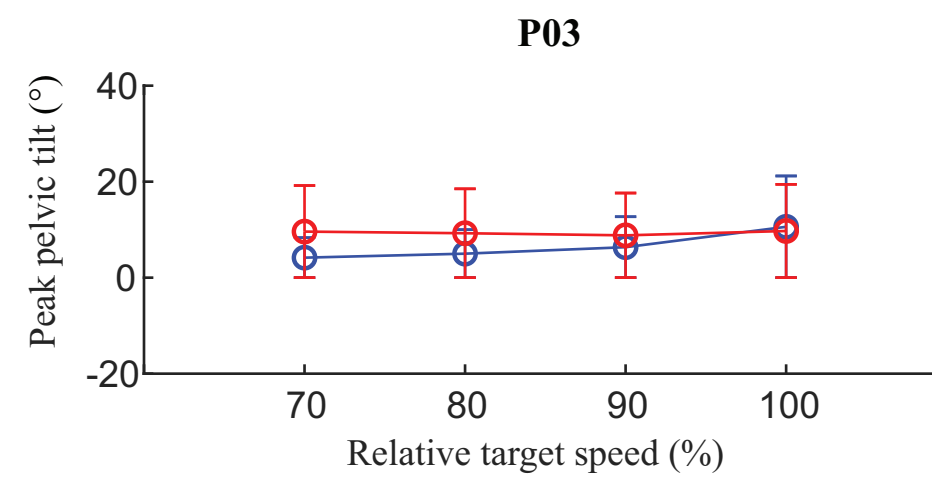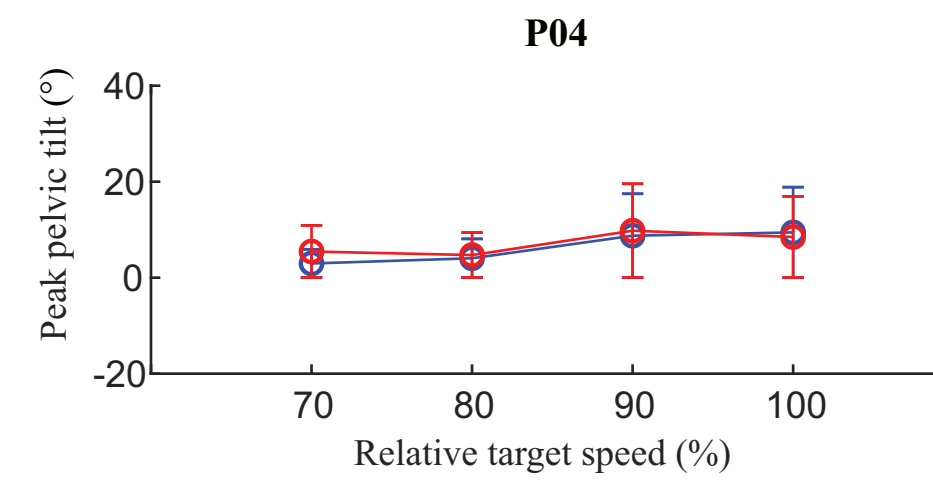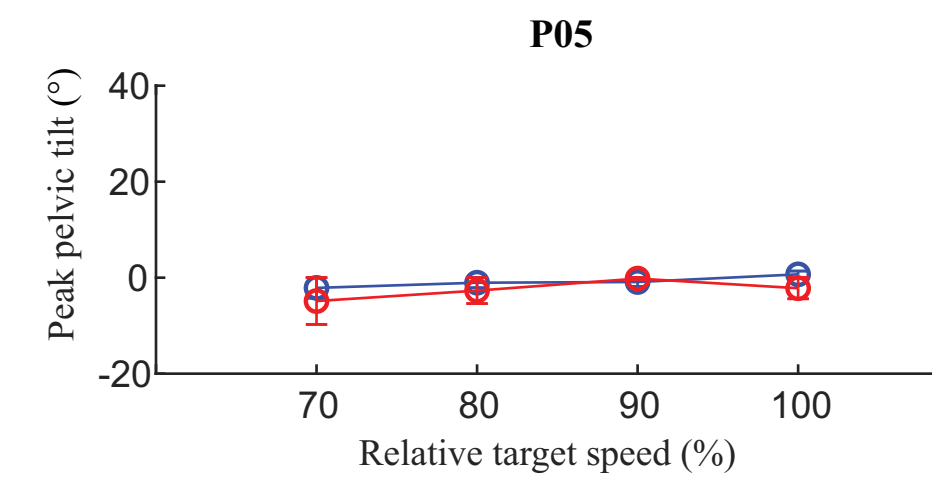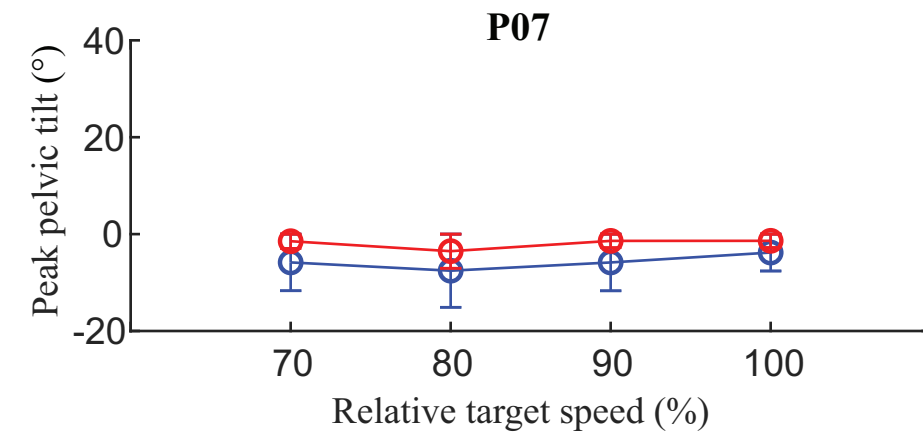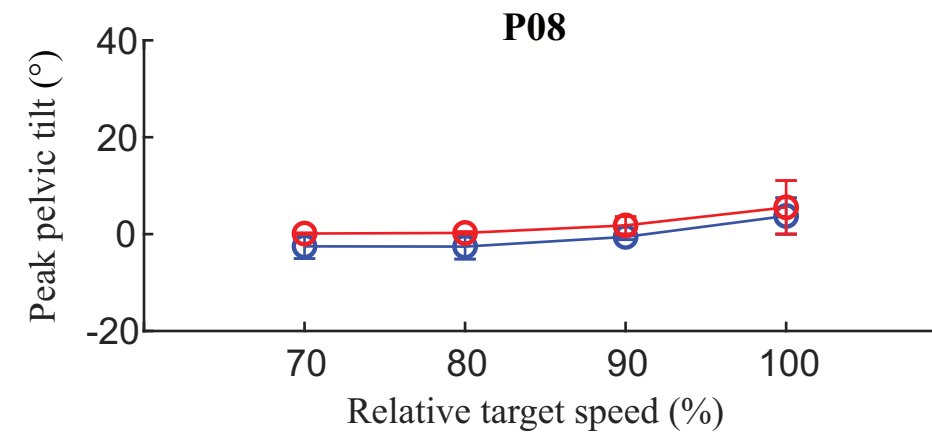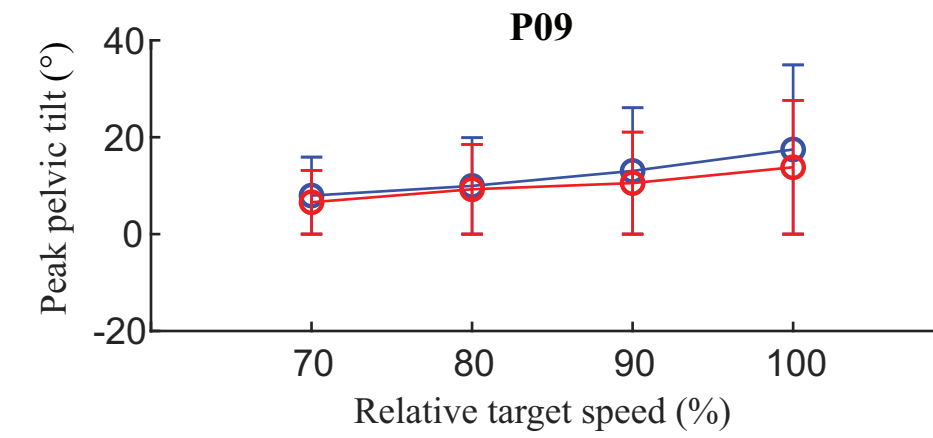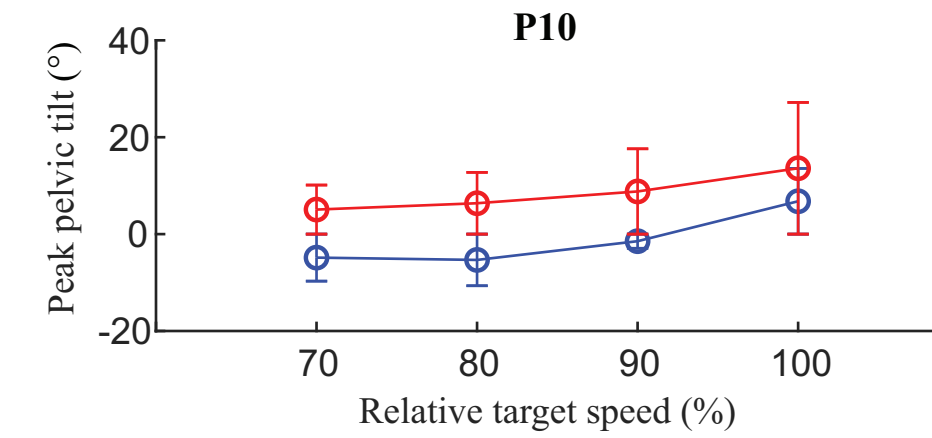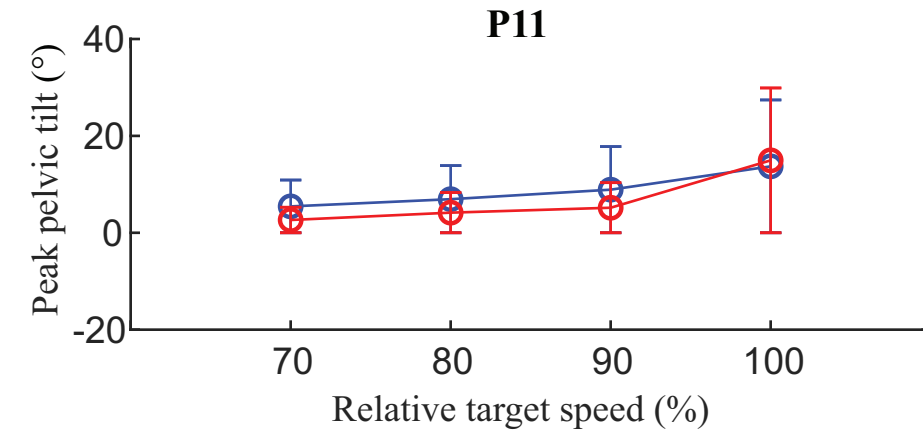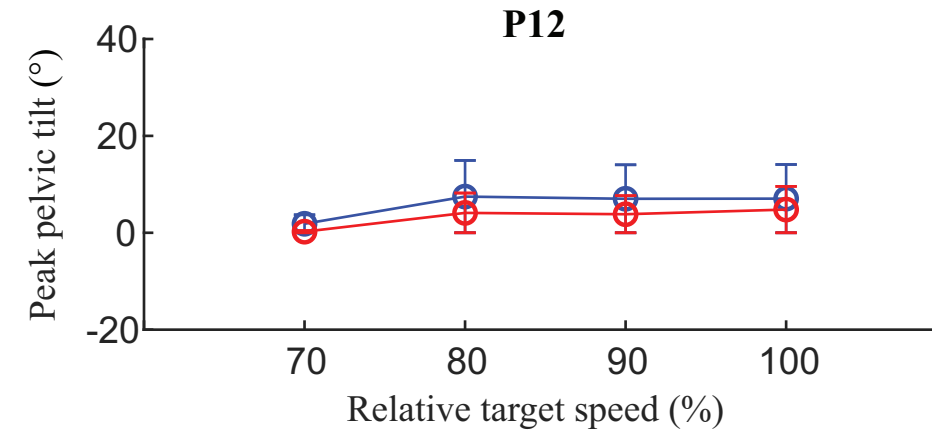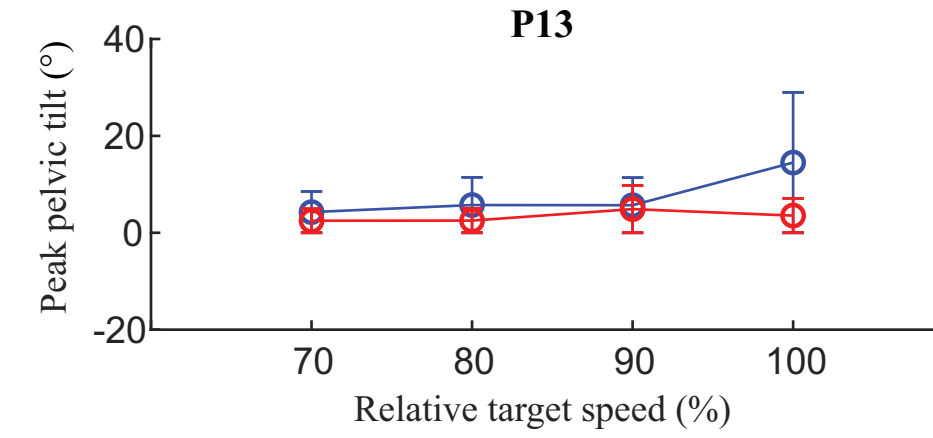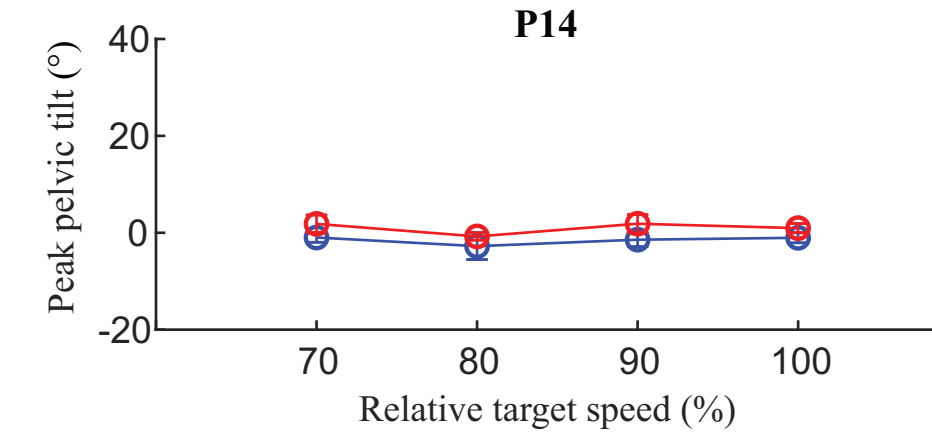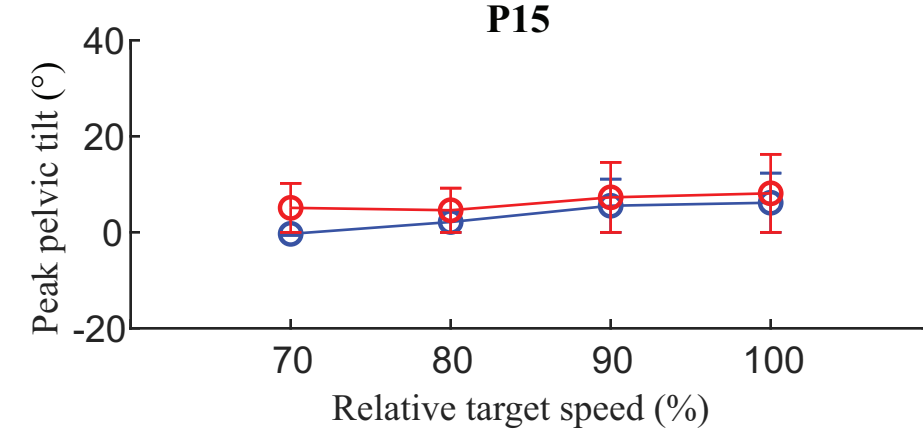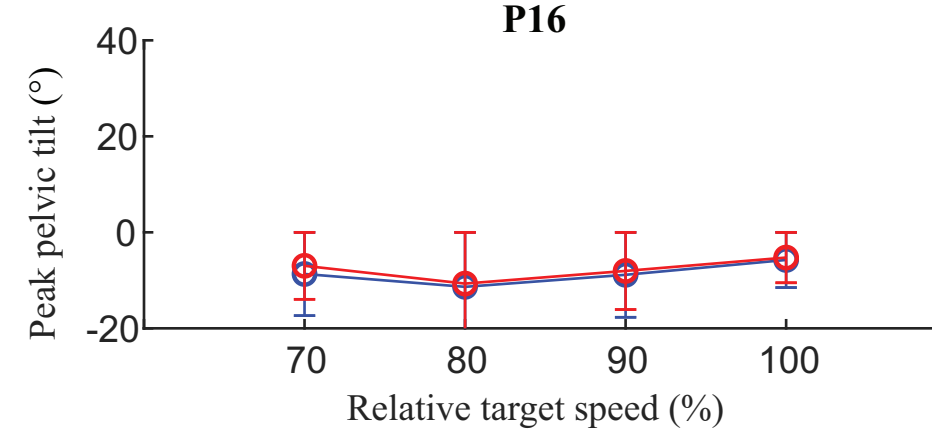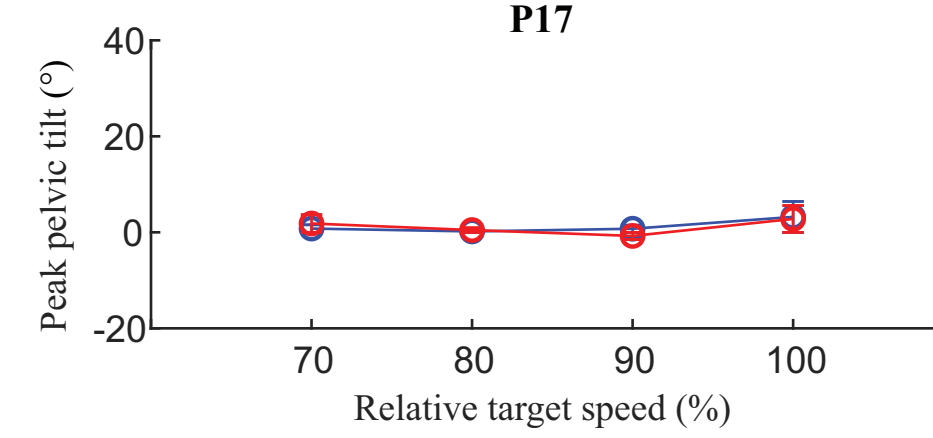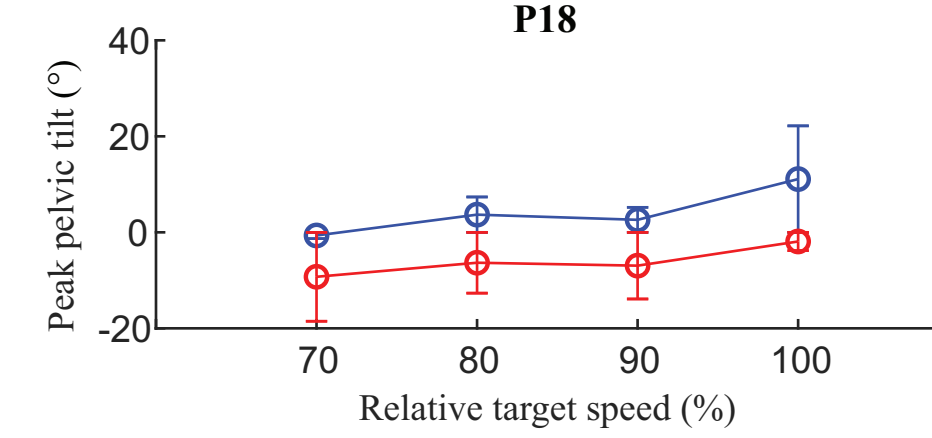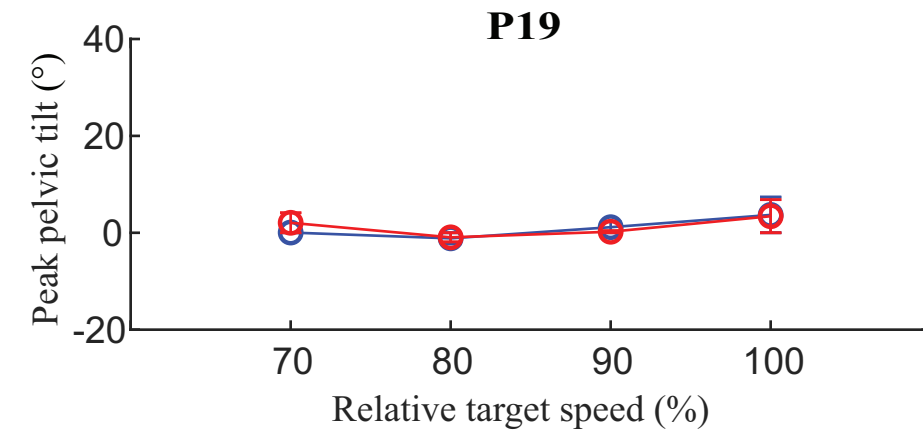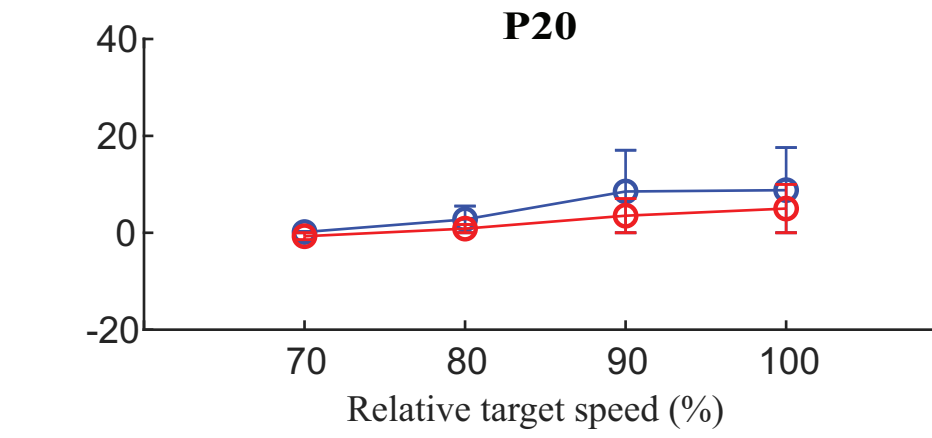

Supplement: Supplementary file 1 [file sensors-23-09599-s001.zip › FigS4_PeakPelvicTiltTrend.pdf]

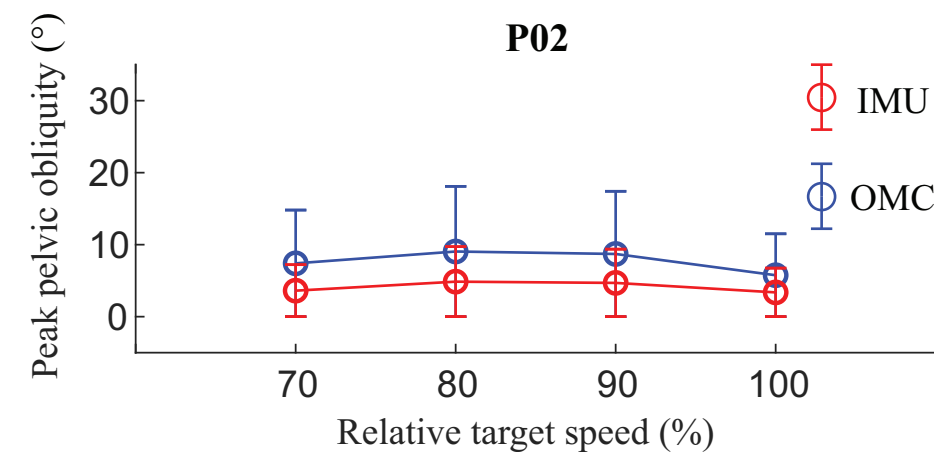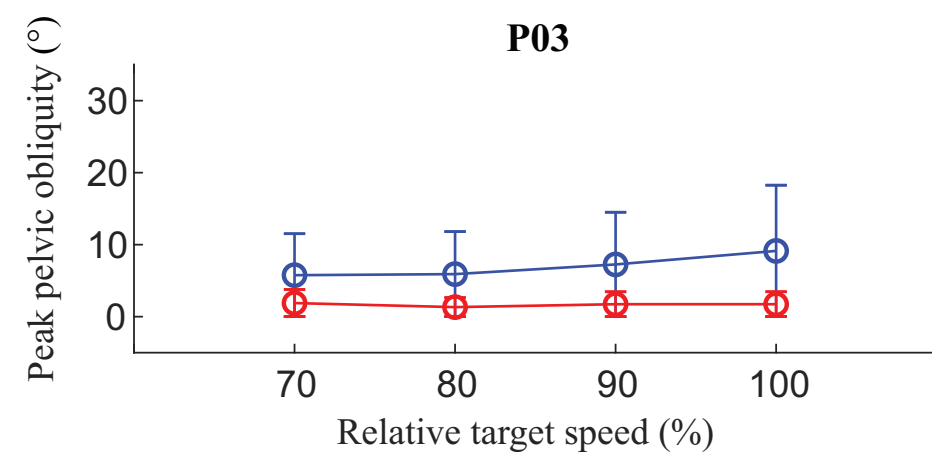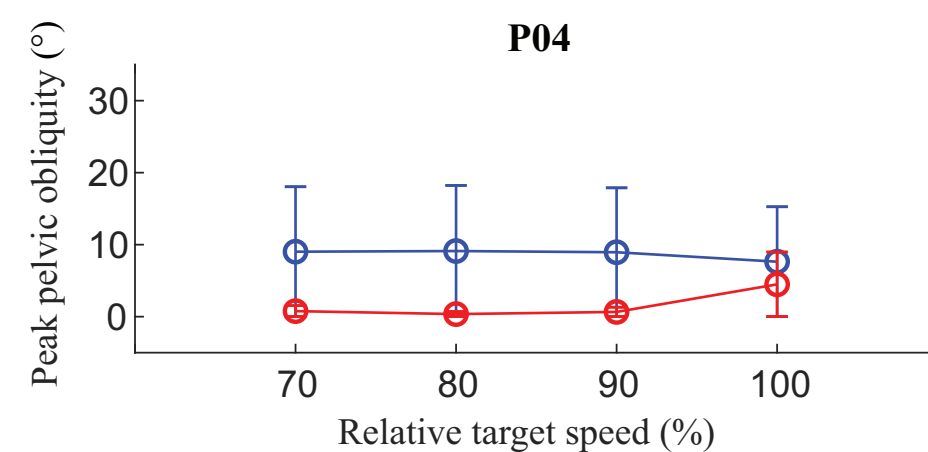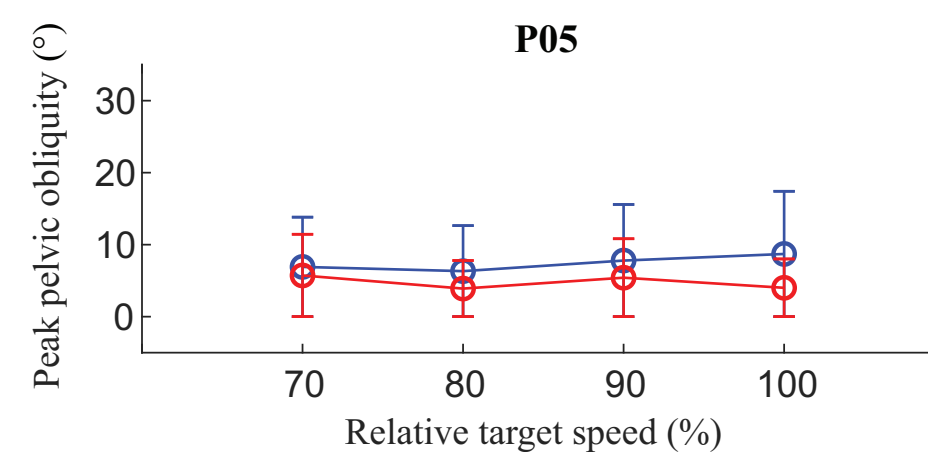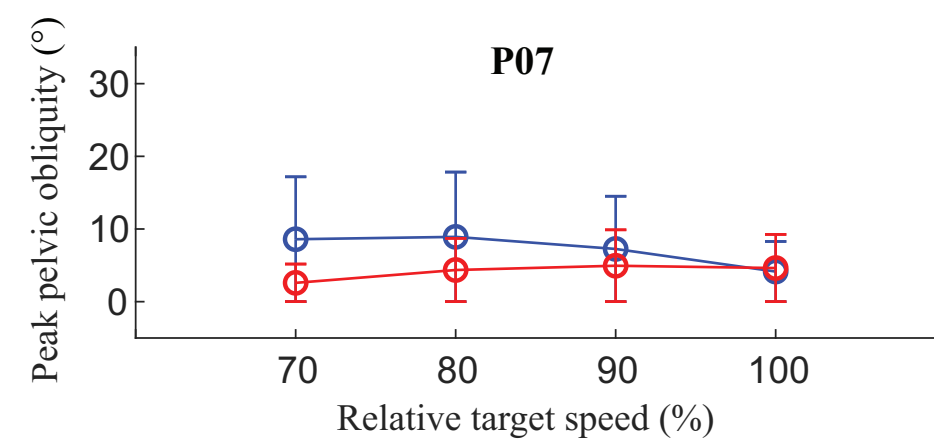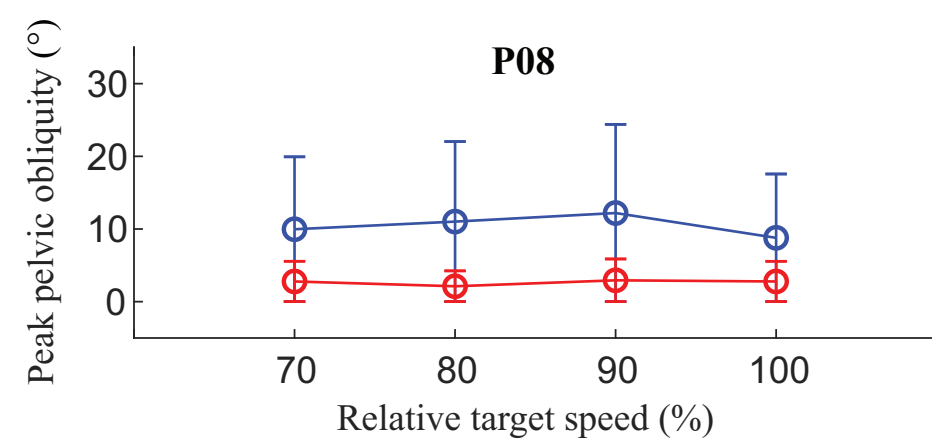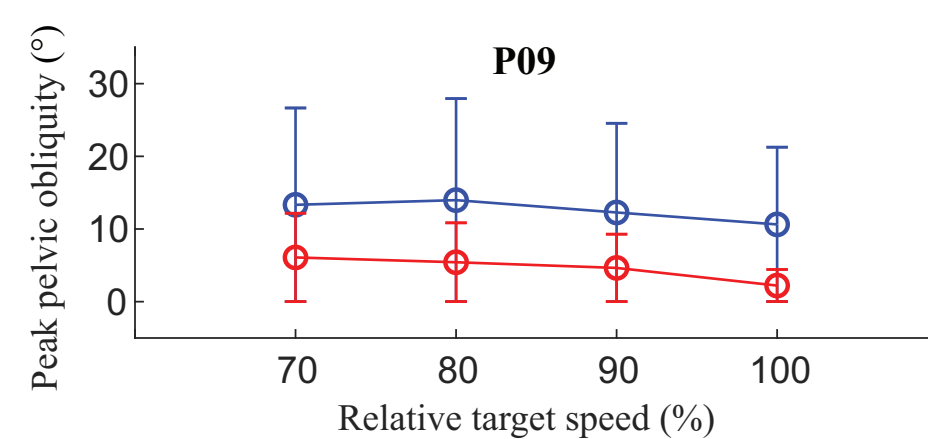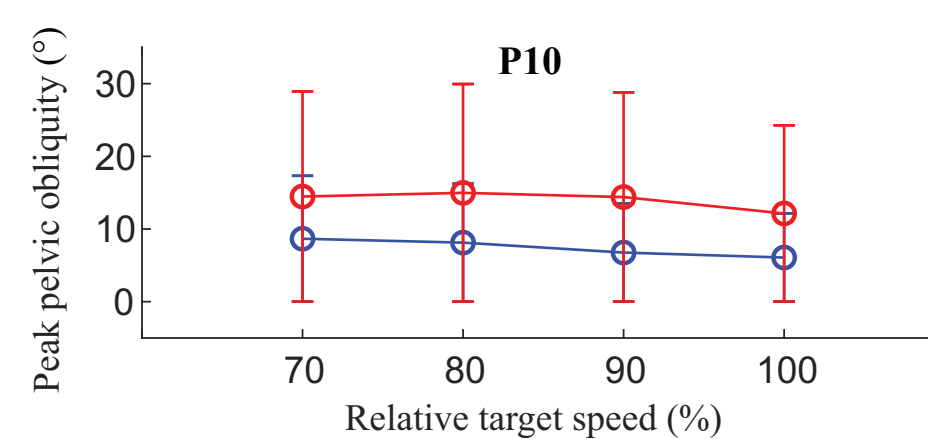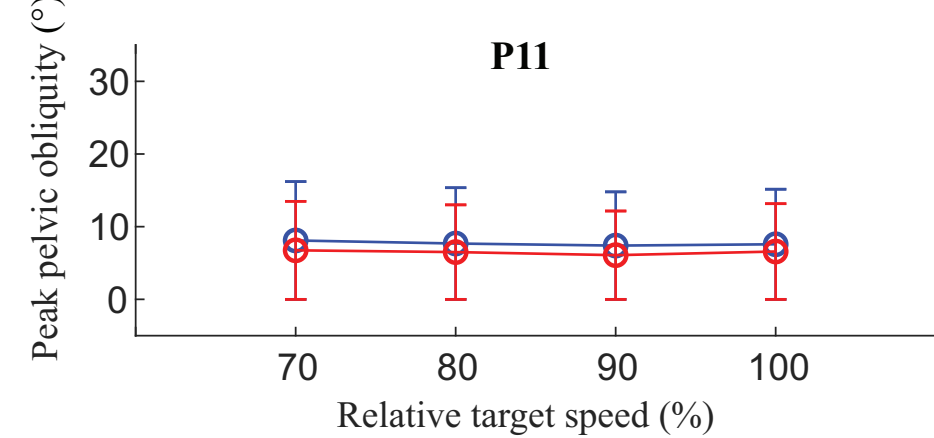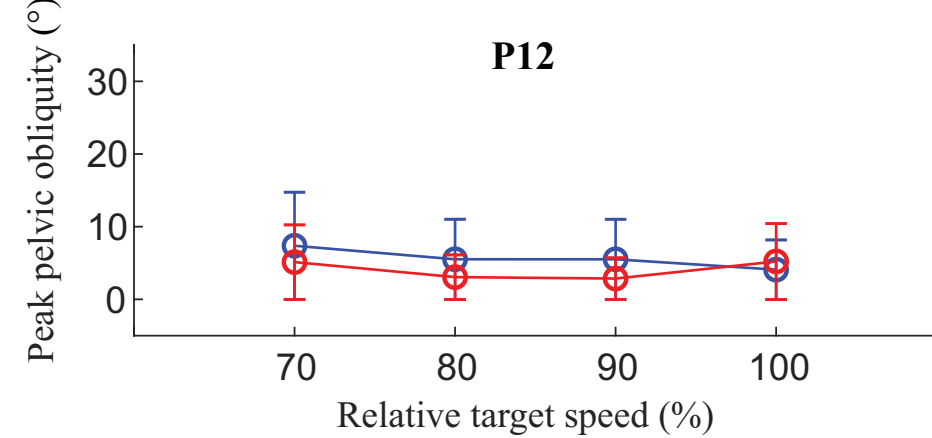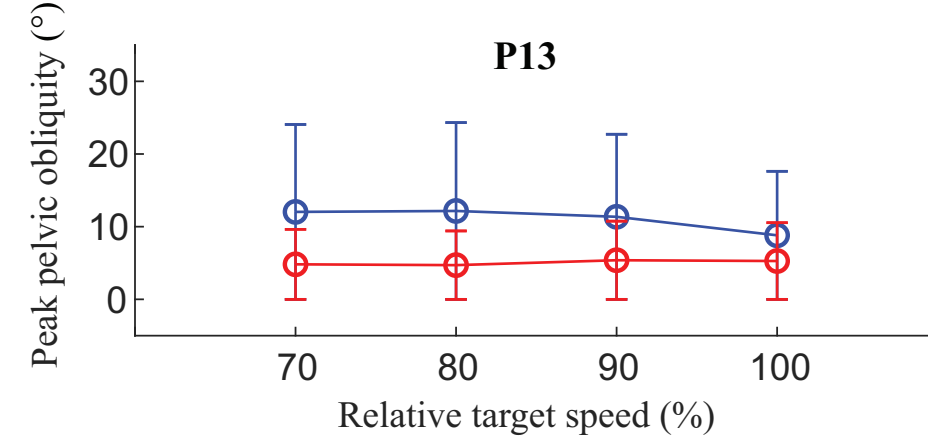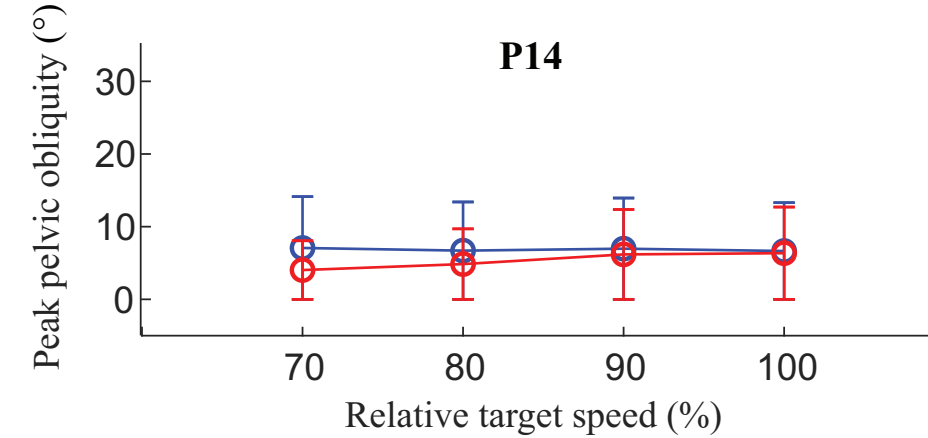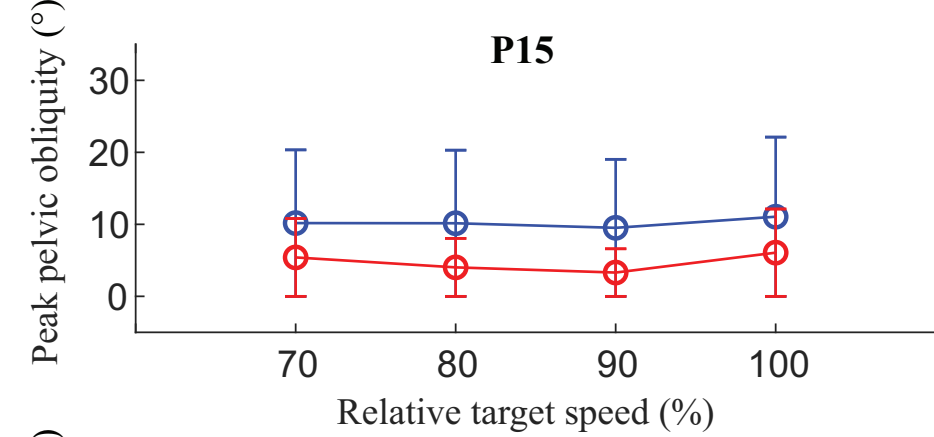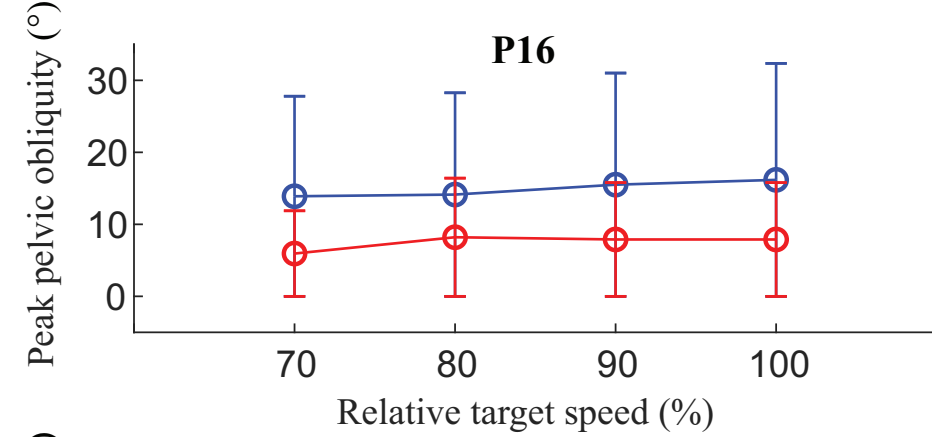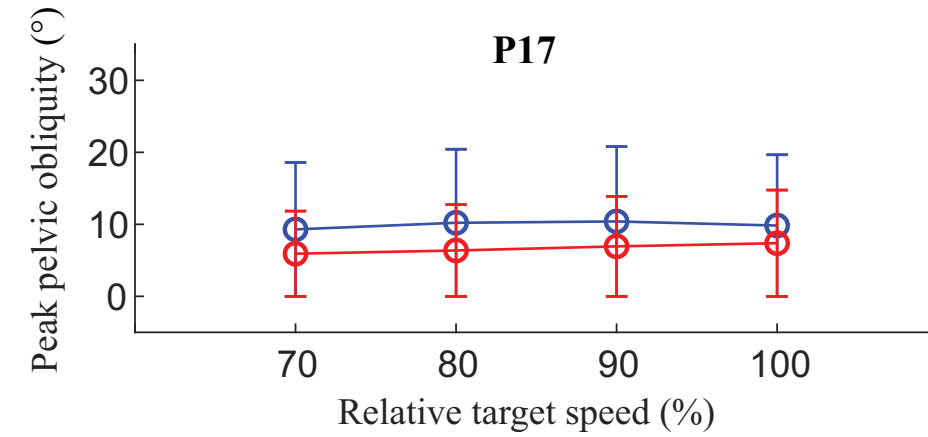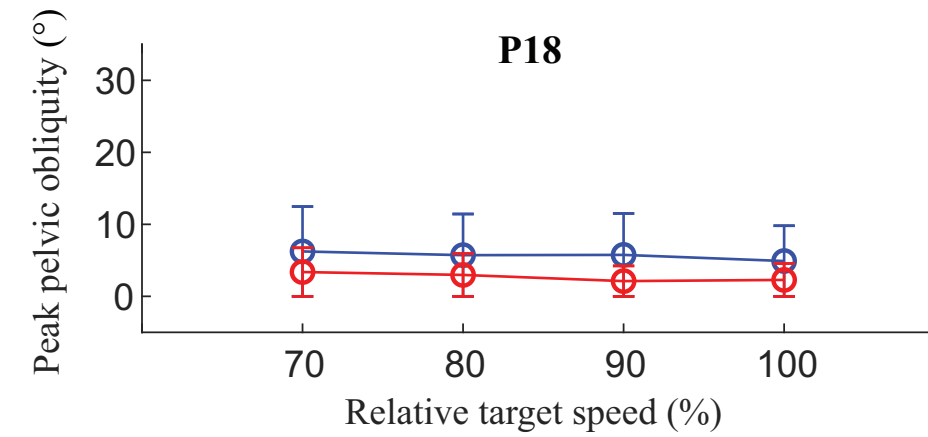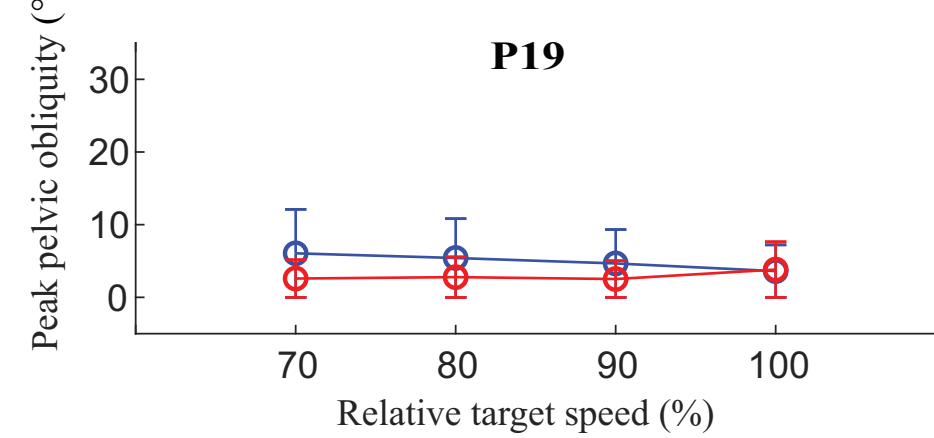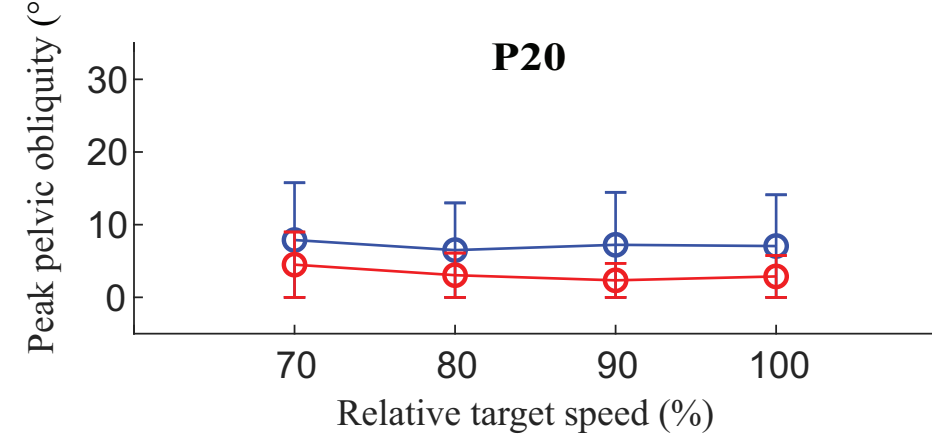

Supplement: Supplementary file 1 [file sensors-23-09599-s001.zip › FigS5_PeakPelvicObliquityTrend.pdf]

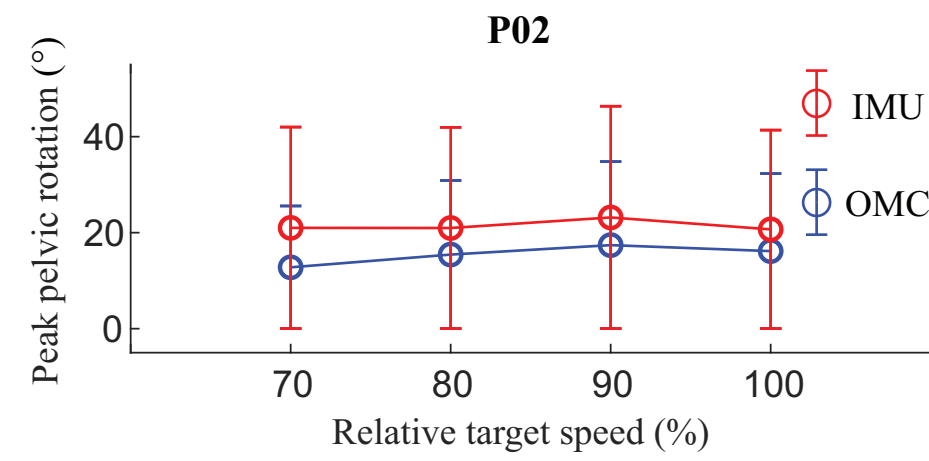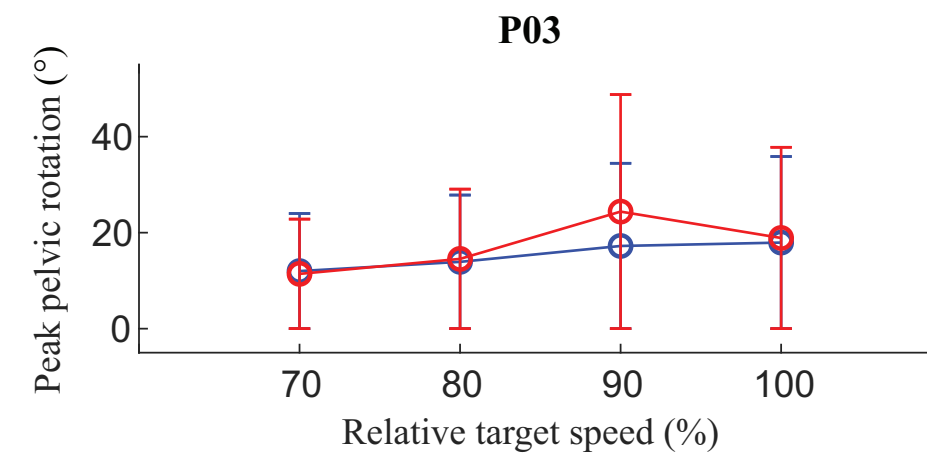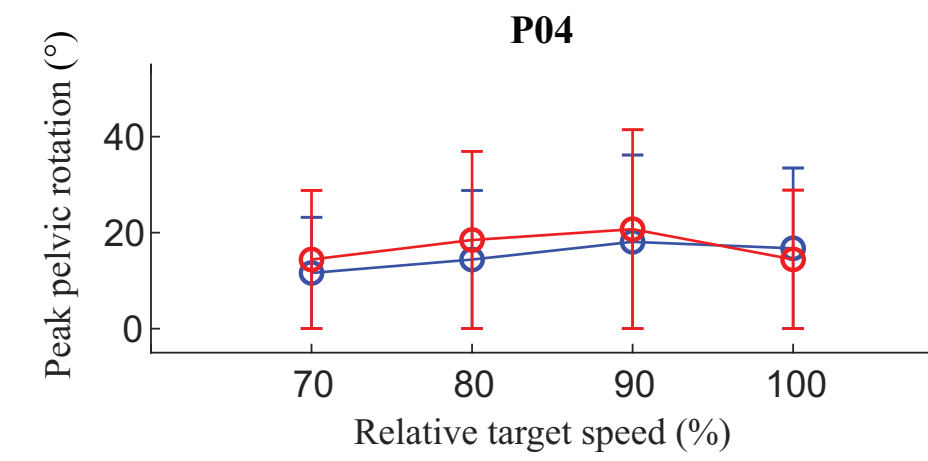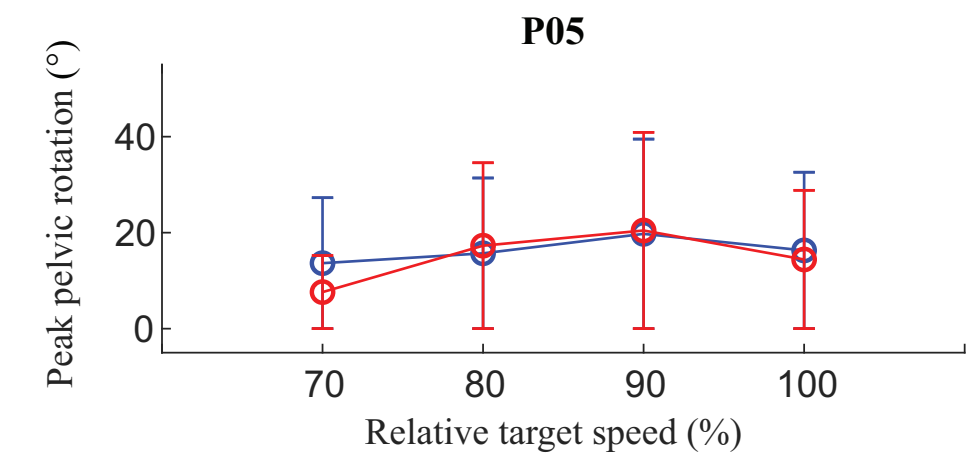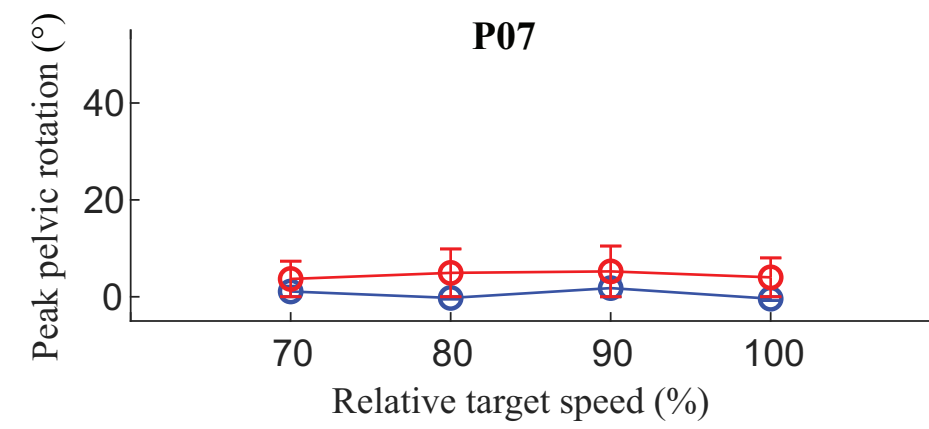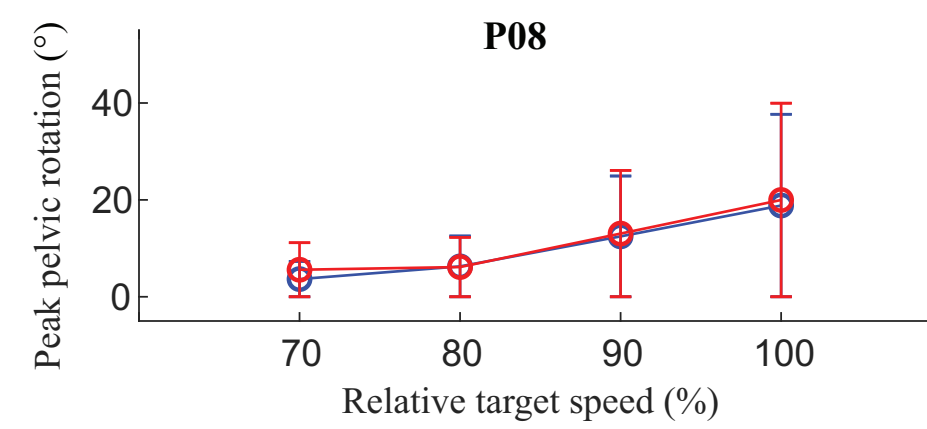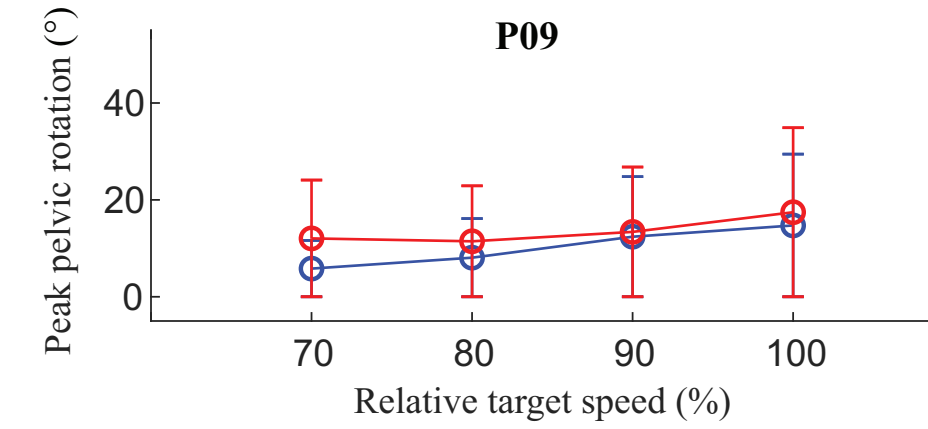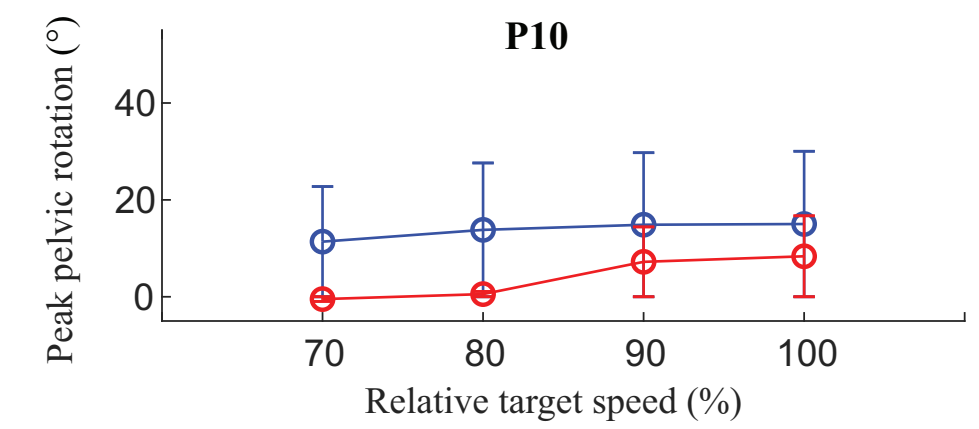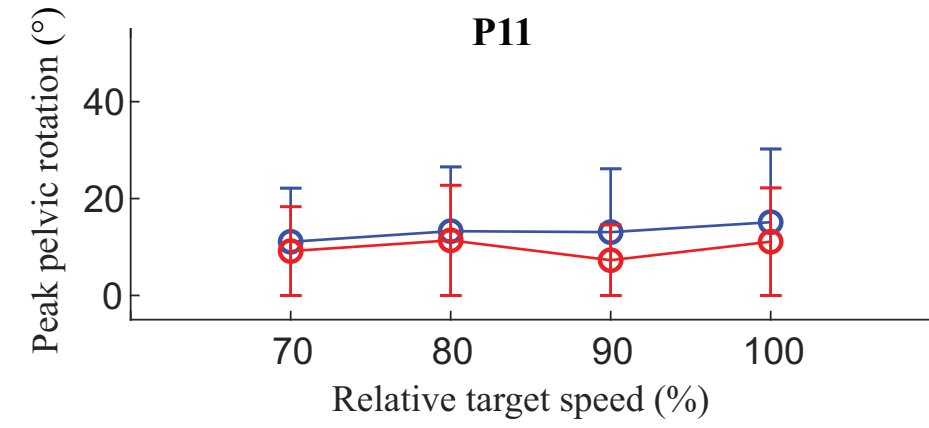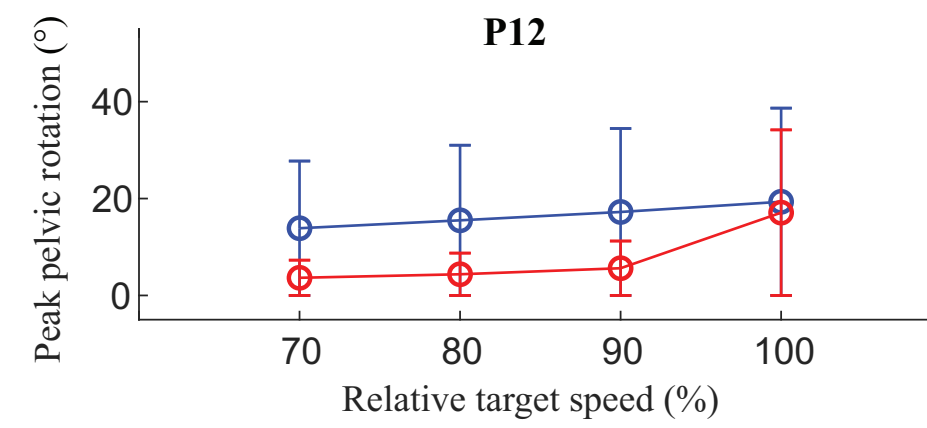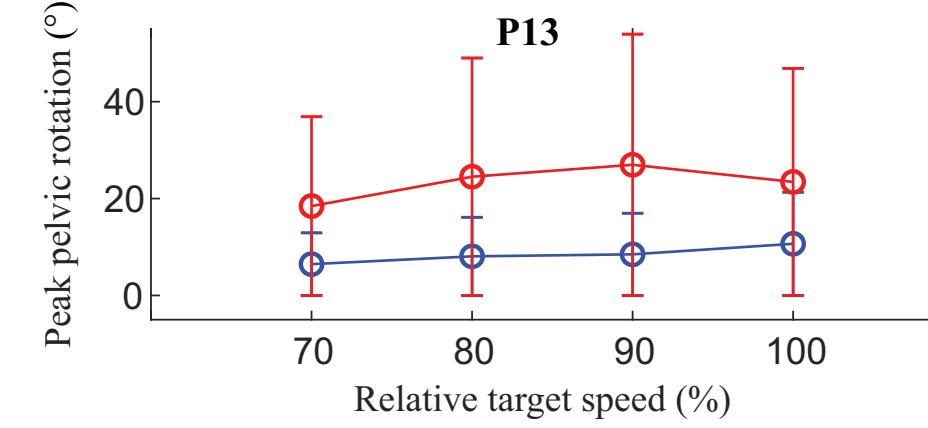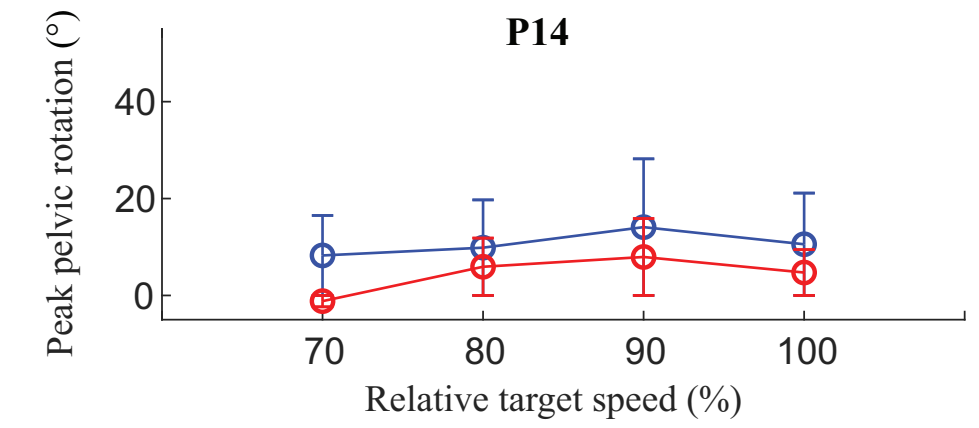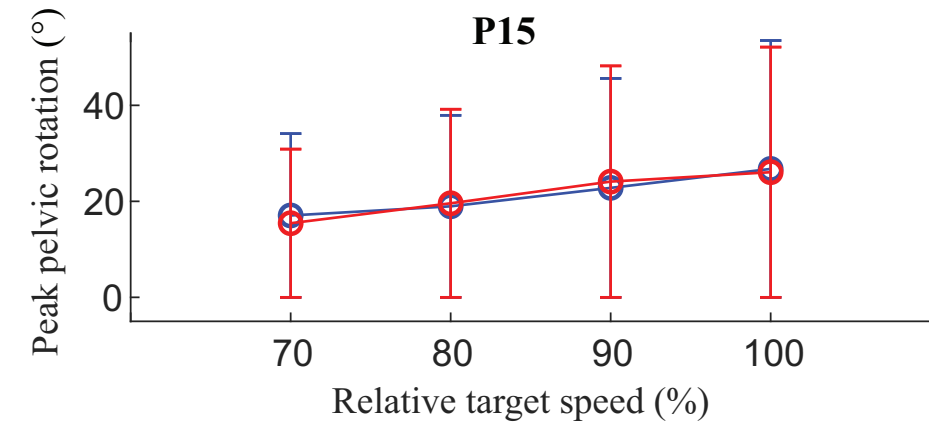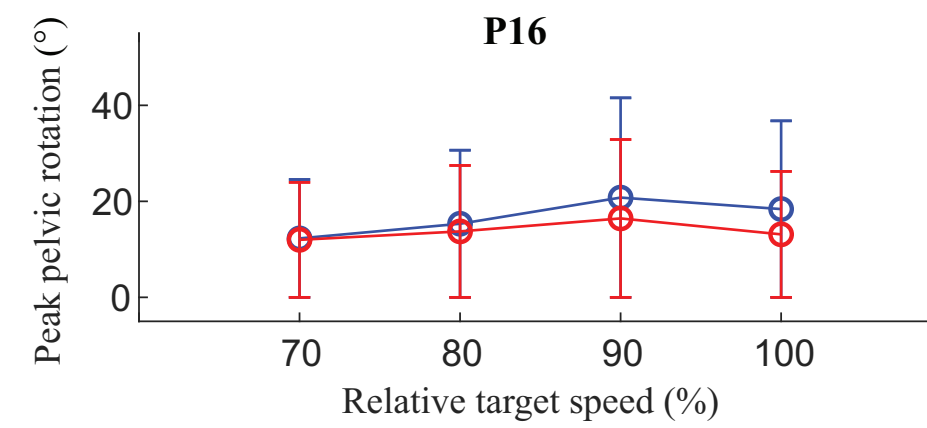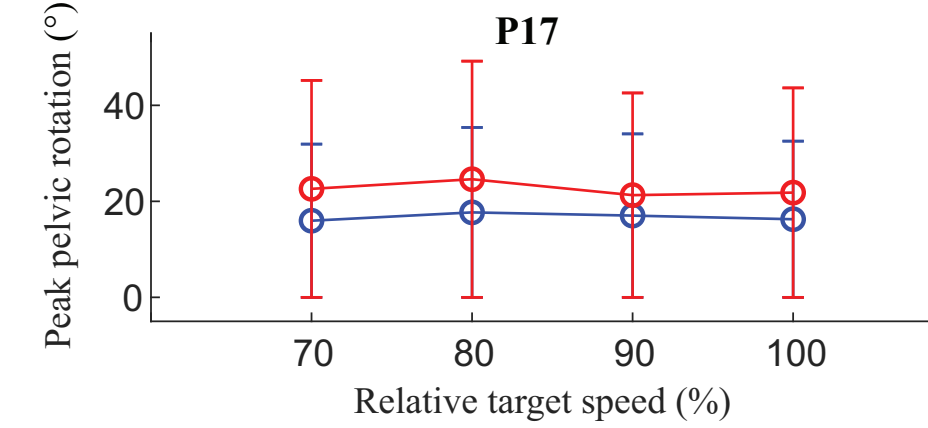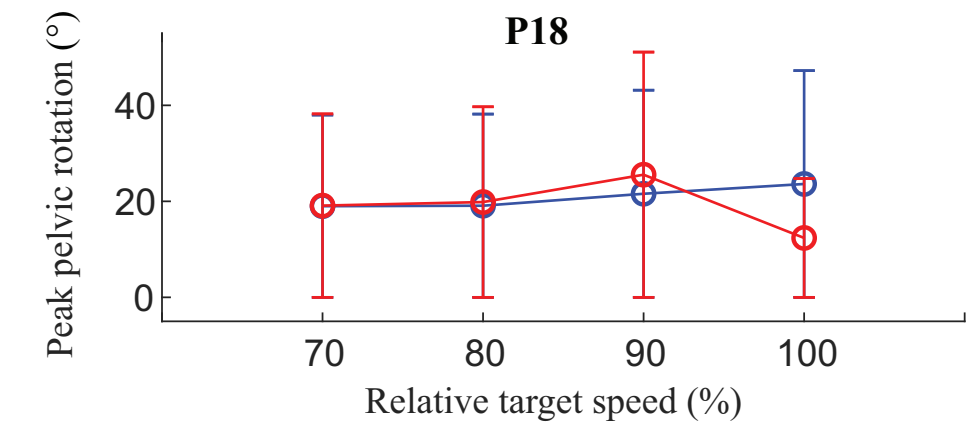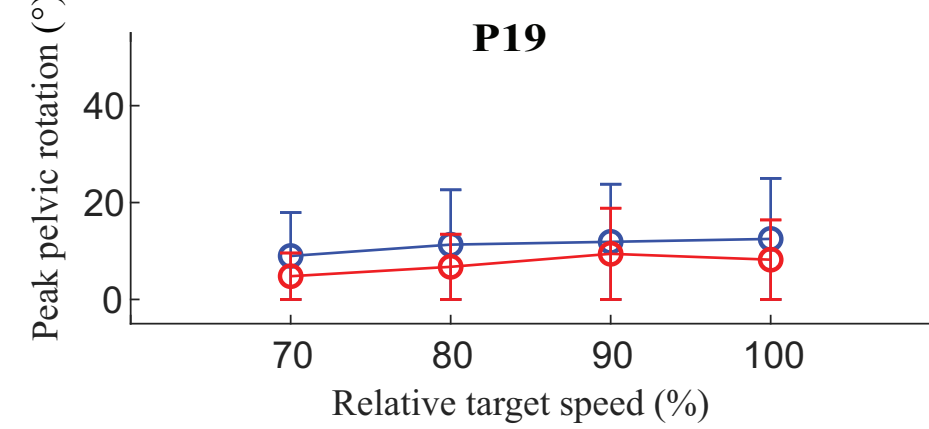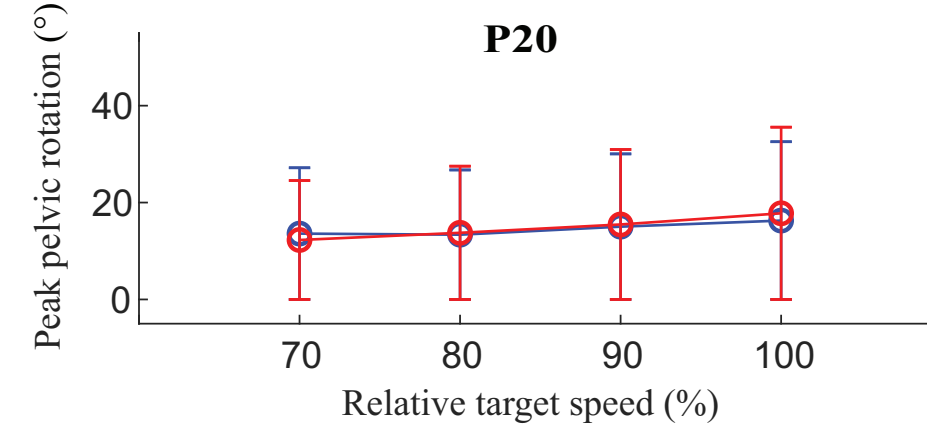

Supplement: Supplementary file 1 [file sensors-23-09599-s001.zip › FigS6_PeakPelvicRotTrend.pdf]
